# Remodeling of the Purkinje Network in Congestive Heart Failure in the Rabbit

**DOI:** 10.1161/CIRCHEARTFAILURE.120.007505

**Published:** 2021-07-12

**Authors:** Sunil Jit R.J. Logantha, Xue J. Cai, Joseph Yanni, Caroline B. Jones, Robert S. Stephenson, Luke Stuart, Gillian Quigley, Oliver Monfredi, Shu Nakao, Il-Young Oh, Tobias Starborg, Ashraf Kitmitto, Akbar Vohra, Robert C. Hutcheon, Antonio F. Corno, Jonathan C. Jarvis, Halina Dobrzynski, Mark R. Boyett, George Hart

**Affiliations:** 1 Division of Cardiovascular Sciences (S.J.R.J.L., X.J.C., J.Y., L.S., G.Q., S.N., I.-Y.O., A.K., A.V., H.D., M.R.B., G.H.), University of Manchester, United Kingdom.; 2 Wellcome Centre for Cell Matrix Research (T.S.), University of Manchester, United Kingdom.; 3 Liverpool Centre for Cardiovascular Science and Department of Cardiovascular and Metabolic Medicine (S.J.R.J.L.), University of Liverpool, United Kingdom.; 4 Division of Clinical Sciences (R.C.H.), University of Liverpool, United Kingdom.; 5 Alder Hey Children’s National Health Service Foundation Trust, Liverpool, United Kingdom (C.B.J.).; 6 School of Sport and Exercise Sciences, Liverpool John Moores University, United Kingdom (R.S.S., J.C.J.).; 7 Institute of Clinical Sciences, University of Birmingham, United Kingdom (R.S.S.).; 8 Manchester University NHS Foundation Trust, United Kingdom (L.S.).; 9 Division of Cardiovascular Medicine, University of Virginia, Charlottesville (O.M.).; 10 Laboratory of Cardiovascular Medicine, National Institute on Aging, NIH Biomedical Research Center, Baltimore, MD (O.M.).; 11 Department of Biomedical Sciences, College of Life Sciences, Ritsumeikan University, Kyoto, Japan (S.N.).; 12 Department of Internal Medicine, Seoul National University Bundang Hospital, Republic of Korea (I.-Y.O.).; 13 Memorial Hermann Children’s Hospital, University of Texas Health, Houston (A.F.C.).; 14 Department of Anatomy, Jagiellonian University, Medical College, Cracow, Poland (H.D.).

**Keywords:** electron microscopy, heart failure, ion channels, Purkinje fibers, rabbits, tomography

## Abstract

**Background:**

Purkinje fibers (PFs) control timing of ventricular conduction and play a key role in arrhythmogenesis in heart failure (HF) patients. We investigated the effects of HF on PFs.

**Methods:**

Echocardiography, electrocardiography, micro-computed tomography, quantitative polymerase chain reaction, immunohistochemistry, volume electron microscopy, and sharp microelectrode electrophysiology were used.

**Results:**

Congestive HF was induced in rabbits by left ventricular volume- and pressure-overload producing left ventricular hypertrophy, diminished fractional shortening and ejection fraction, and increased left ventricular dimensions. HF baseline QRS and corrected QT interval were prolonged by 17% and 21% (mean±SEMs: 303±6 ms HF, 249±11 ms control; n=8/7; *P*=0.0002), suggesting PF dysfunction and impaired ventricular repolarization. Micro-computed tomography imaging showed increased free-running left PF network volume and length in HF. mRNA levels for 40 ion channels, Ca^2+^-handling proteins, connexins, and proinflammatory and fibrosis markers were assessed: 50% and 35% were dysregulated in left and right PFs respectively, whereas only 12.5% and 7.5% changed in left and right ventricular muscle. Funny channels, Ca^2+^-channels, and K^+^-channels were significantly reduced in left PFs. Microelectrode recordings from left PFs revealed more negative resting membrane potential, reduced action potential upstroke velocity, prolonged duration (action potential duration at 90% repolarization: 378±24 ms HF, 249±5 ms control; n=23/38; *P*<0.0001), and arrhythmic events in HF. Similar electrical remodeling was seen at the left PF-ventricular junction. In the failing left ventricle, upstroke velocity and amplitude were increased, but action potential duration at 90% repolarization was unaffected.

**Conclusions:**

Severe volume- followed by pressure-overload causes rapidly progressing HF with extensive remodeling of PFs. The PF network is central to both arrhythmogenesis and contractile dysfunction and the pathological remodeling may increase the risk of fatal arrhythmias in HF patients.

## Abstract 2

Supplemental Digital Content is available in the text.

**WHAT IS NEW?**

- Surgically induced aortic regurgitation followed by abdominal aortic banding in rabbits induced severe congestive heart failure with ascites, pleural or pericardial effusions, enlarged dilated hearts, reduced ejection fraction, and conduction system dysfunction, all clinical features of patients with heart failure.
- Discordance between the extent of the changes in the Purkinje network compared to those in the ventricular muscle with marked structural, molecular, and electrical remodeling and arrhythmic events in the Purkinje network in heart failure.
- Two-fold increases in action potential duration in the free-running Purkinje fiber and the Purkinje-ventricular junction but no change proximally in the left bundle branch and in the left ventricle.

**WHAT ARE THE CLINICAL IMPLICATIONS?**

- Dysfunction of the Purkinje network and left bundle branch block are seen in heart failure. Here, we report the basis of conduction slowing and the mechanism of dysfunction and identify the arrhythmic substrate in the distal Purkinje network.
- Anticipation of left ventricular dilatation and strategies to optimize hemodynamics and reduce afterload may reduce the left ventricular dilatation and Purkinje fiber remodeling and consequent conduction abnormalities.
- His-bundle pacing is unlikely to help in the face of severe dysfunction of the distal Purkinje network.

Heart failure (HF) is a progressive disease in which death occurs through arrhythmias and pump failure.^1,2^ The His-Purkinje network with its arborized architecture plays a central role in ventricular activation and comprises the His-bundle, its subdivisions the right and left bundle branches (LBB), and the terminal Purkinje fibers (PF), facilitating rapid and synchronous ventricular activation.^3^ Twenty-six percent of systolic HF patients have LBB block (LBBB).^4^ LBBB results in ventricular dyssynchrony, reduced cardiac output, and is associated with increased mortality in congestive HF.^5^ Sudden death in HF patients can be the result of ventricular tachyarrhythmias including fibrillation.^6,7^ Our hypothesis is that HF results in dysfunction of the ventricular conduction system, particularly the PFs causing ventricular dyssynchrony and tachyarrhythmias.

PFs are longitudinal assemblies of Purkinje cells wrapped in sleeves of insulating connective tissue.^3,8^ They have specialized membrane electrical properties, eg, fast action potential upstroke velocity (dV/dt_max_) and high action potential amplitude compared with ventricular myocytes, resulting in fast action potential conduction.^8^ Fast conduction is also facilitated by strong electrical coupling between cells—PFs express large-conductance Cx (connexins), Cx40 and Cx43.^3,9,10^ In Cx40 deficient mice, conduction in the LBB is impaired and there is right bundle branch block.^11^ In PFs, the early repolarization of the action potential (phase-1) is more pronounced, the plateau potential is more negative and the action potential duration (APD) and refractoriness are longer than those of ventricular muscle.^8,12^ PFs show automaticity, but rapid excitation during sinus rhythm inhibits automaticity in PFs by overdrive suppression.^13^ However in the absence of sinus rhythm, HCN (hyperpolarization-activated cyclic nucleotide-gated) channels responsible for the funny current (*I*_f_) can drive pacemaking.^14^ Furthermore, Ca^2+^ release from the sarcoplasmic reticulum can elicit spontaneous Ca^2+^ waves and delayed afterdepolarizations resulting in automaticity.^15^

Small animal models provide an opportunity to investigate remodeling over a convenient time frame and the rabbit has several advantages over the mouse and rat: it is more similar to human in respect of in vivo heart rate, action potential configuration, Ca^2+^-handling, contractile protein expression, and left ventricular (LV) mechanics.^16–18^ The cardiac conduction system in rabbit is well-characterized, providing a basis for investigating changes in disease.^3,8,19–21^ The extensive PF network traversing rabbit ventricular chambers enables structural, molecular, and functional investigations with minimal contamination from other cell types.^3,22^ Here, we have used a well-established rabbit model of volume- and pressure-overload that is prone to ventricular tachycardia^23^ to investigate PF remodeling in HF. We demonstrate widespread dysfunction of the PF network and show that the PF network is notably more affected than the ventricular myocardium.

## Methods

### Rabbit HF Model

Congestive HF was induced in male New Zealand white rabbits (n=23; sham-operated controls, n=21) weighing 2.5 to 3 kg, age ≈3 months (B&K Ltd, United Kingdom), as previously described.^24^ Briefly, severe incompetence of the aortic valve was induced with a catheter introduced via the right carotid artery, followed after 3 weeks by abdominal aortic constriction at the level of the renal arteries. This work was performed under the terms of the Animals (Scientific Procedures) Act (1986 and subsequent amendments) and UK Home Office project licenses PPL 40/3135 and 40/3689. All supporting data are available within the article and its Data Supplement.

### Echocardiography and ECG

HF progression was assessed by 2-dimensional echocardiography (GE Vivid3, 5 MHz transducer) in conscious rabbits, before and after surgery. Rabbits were anaesthetized with 50 mg/kg ketamine and 2% isoflurane and 3-lead ECGs were recorded for ≈2000 consecutive beats at baseline and after pharmacological autonomic block. Animals were humanely killed at ≈5 weeks after aortic constriction, by pentobarbitone sodium overdose, followed by vertical sternotomy and removal of the heart into oxygenated (95% O_2_–5% CO_2_) Tyrode solution (in mM: NaCl, 120; KCl, 4; MgSO_4_.7H_2_O, 1.3; NaH_2_PO_4_.2H_2_O, 1.2; CaCl_2_, 1.2; NaHCO_3_, 25.2; Glucose, 5.8; pH 7.4).

### High-Resolution X-Ray Computed Tomography (Micro-Computed Tomography Imaging)

Hearts were perfusion fixed in situ, stained using an iodine-based contrast solution^22,25^ and micro-computed tomography (CT) scanned using a custom bay Nikon 320 kV scanner within the Henry Moseley X-ray Imaging Facility at the University of Manchester. PF networks were segmented from the data using a 3-dimensional segmentation technique and tissue volumes and free-running lengths were calculated.

### Quantitative Polymerase Chain Reaction and Immunohistochemistry

Total RNA was isolated from frozen samples using a modified Qiagen fibrous tissue protocol with a DNase digestion step. Briefly, total RNA (150 ng) was reverse-transcribed with Superscript III reverse transcriptase (Invitrogen) in 20 μL reactions according to manufacturer’s instructions using random hexamer priming. Aliquots of resulting cDNA were diluted 10-fold in RNase-free water for quantitative polymerase chain reaction. mRNA and cDNA samples from individual rabbits were kept separate and not pooled.

The relative content of selected cDNA fragments was determined, in triplicate at least, with quantitative polymerase chain reaction in 1 μL aliquots of cDNA using Applied Biosystems 7900HT Real-Time PCR system and detection with SYBR green (Applied Biosystems) in 10 μL reactions. Primer sequences and annealing temperatures have been described previously.^19,26^ All runs were 40 cycles in duration. To measure accurately the abundance of a selected cDNA in different tissues a double standardization method (modified ΔΔCT method) was used. Each polymerase chain reaction run included a calibrator sample (cDNA sample containing equal mixture of all tissue types). Abundance of a selected cDNA in a sample was expressed as a ratio of its abundance in the calibrator sample. Abundance of housekeeper cDNA, 28S, was similarly calculated. The first standardization allowed for variations between runs. Then the calculated abundance ratio of the selected cDNA was again expressed as ratio of the calculated abundance ratio for 28S. Frozen hearts were cryosectioned (20-μm thickness) and transferred onto glass slides for investigating protein expression by immunohistochemistry.^26–28^

### Electrophysiology

Intracellular action potentials were recorded using sharp microelectrodes as described previously.^28^ Briefly, glass microelectrodes (20–40 MΩ resistance) were backfilled with 3M KCl and used to record intracellular action potentials in tissue preparations. Signals acquired at 20 kHz were amplified, digitized, and stored on a computer. Action potential measurements were made using LabChart 8 software (AD Instruments).

### Statistical Analysis

Data are expressed as mean±SEM. Student unpaired *t* test was used for datasets with 2 groups. Welch correction was applied to groups with unequal variances. For ≥3 groups 2-way ANOVA for independent/interacting effects of disease and autonomic block/tissue, followed by Sidak multiple comparison post hoc test was used. When post hoc adjusted 0.05<*P*<0.1, *t* testing of respective HF versus control datasets was performed and significant differences were reported. *P*≤0.05 was deemed statistically significant.

Further details of the methods used are given in the Data Supplement.

## Results

### Congestive HF and Cardiac Conduction System Disease

**Figure 1.**
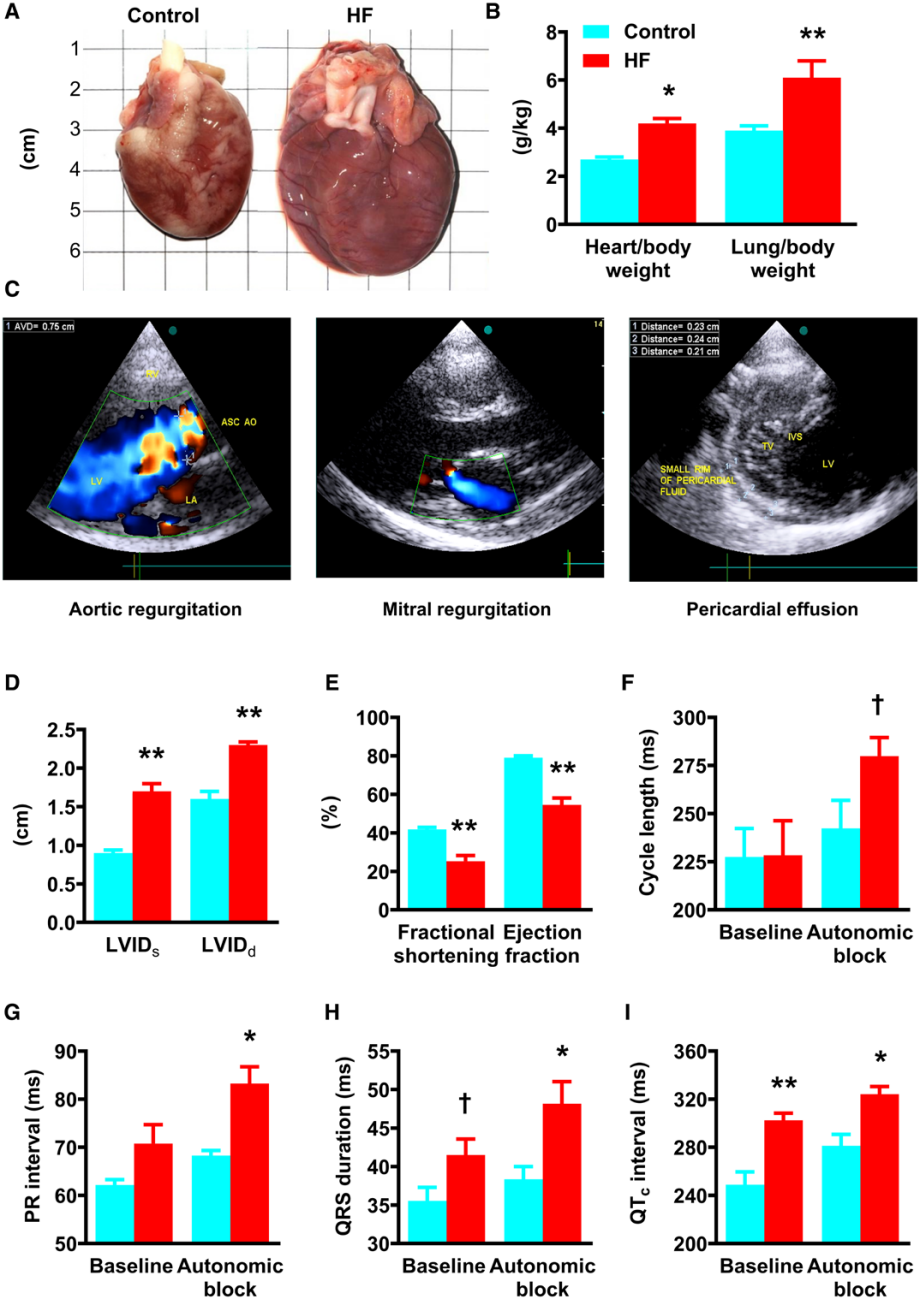
**Cardiac morphometric and functional assessments in the rabbit model of volume and pressure-overload–induced congestive heart failure (HF)**. **A**, Images of typical control and HF rabbit hearts at termination. **B**, The heart-to-body weight and lung-to-body weight ratios of control (blue) and HF (red) rabbits. **C**, Representative echocardiography images (2-dimensional parasternal views) taken from an HF rabbit. **Left**, Color-flow Doppler showing severe aortic regurgitation. **Center**, color-flow Doppler showing functional mitral regurgitation. **Right**, Small pericardial effusion over the surface of both ventricles. **D** and **E**, Left ventricular (LV) internal diameter in systole (LVID_s_) and diastole (LVID_d_) and fractional shortening and ejection fraction in control and HF rabbits. **F–I**, In vivo conscious ECG parameters: cycle length, PR interval, QRS duration and corrected QT (QT_c_) interval before (baseline) and after pharmacological autonomic blockade. Data are presented as mean±SEMs of control (n=7) and HF (n=8) hearts. IVS indicates interventricular septum. ASC AO indicates ascending aorta; AVD, aortic valve diameter; LA, left atrium; RV, right ventricle; and TV, tricuspid valve. Statistical differences between control and HF rabbits were assessed using 2-way ANOVA followed by Sidak multiple comparison test (*) or *t* test (†); *,†*P*≤0.05; **P≤0.001.

HF rabbits showed evidence of advanced congestive HF with ascites, pleural, or pericardial effusions at termination. Failing hearts were enlarged, and heart/body and lung/body weight ratios were elevated (Figure 1A and 1B; Table I in the Data Supplement). Representative echocardiographic images illustrate LV dilatation and functional mitral regurgitation (Figure 1C). LV dimensions increased progressively (Figure 1D) and, at termination, fractional shortening (25.3±2.9% HF, 41.8±1.1% control) and ejection fraction (54.5±3.6% HF, 79.1±0.9% control) were substantially reduced (Figure 1E). ECG under general anesthesia before termination showed no change in baseline cycle length or PR interval in HF. However, after autonomic blockade, both cycle length (279.9±9.7 ms HF, 242.3±14.6 ms control) and PR interval (83.3±3.5 ms HF, 68.3±1.1 ms control) were prolonged (Figure 1F and 1G), suggesting intrinsic sinus bradycardia and atrioventricular node conduction slowing in HF. The HF rabbits showed prolongation of the QRS duration and corrected QT interval both at baseline and after autonomic blockade (Figure 1H and 1I), indicating slowed PF conduction and delayed ventricular repolarization.

### Micro-CT Imaging

**Figure 2.**
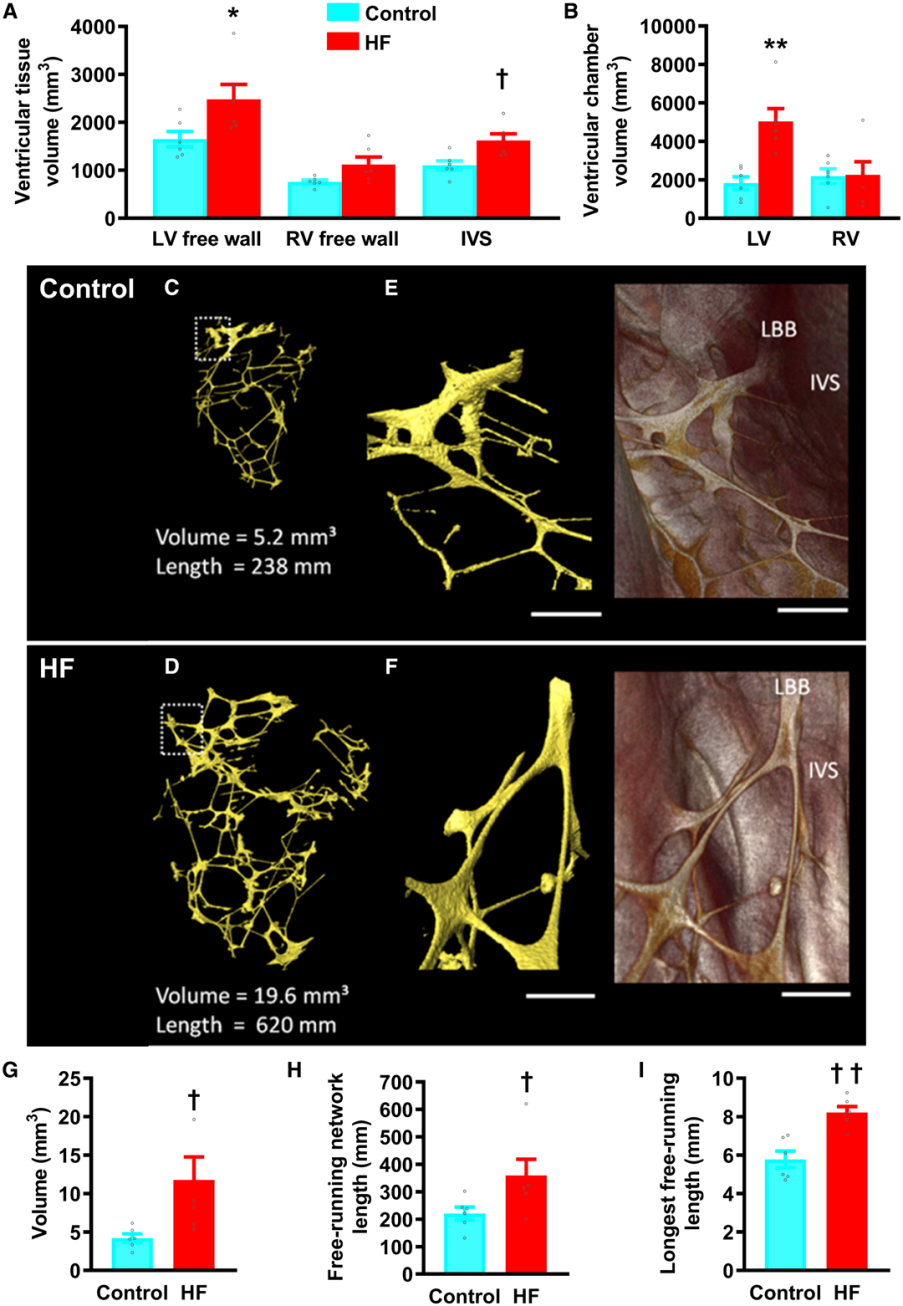
**Structural remodeling of the Purkinje fiber (PF) network in heart failure (HF)**. **A**, Myocardial tissue volumes for left ventricular (LV) and right ventricular (RV) free wall and interventricular septum (IVS). **B**, LV and RV chamber volumes. **C** and **D**, Scaled 3-dimensional (3D) surface renderings of the LV free-running Purkinje network segmented from micro-computed tomography (CT) data in a control (**C**) and failing (**D**) heart; volumes are viewed anteriorly. **E** and **F**, high power 3D surfaces (**left**) and corresponding volume renderings (**right**) showing the most superior-ventral free-running branch of the PF network commencing from the IVS. Scale bars represent 2 mm. **G–I**, Mean tissue volume (**G**), total free-running network length (**H**), and mean longest segment (**I**) of the LV free-running PF network segmented from micro-CT data of control (blue bars) and HF (red bars) samples. Data are presented as mean±SEMs of control (n=6) and HF (n=6) hearts. Statistical differences between control and HF rabbits were assessed using Student unpaired *t* test. LBB indicates left bundle branch. †*P*≤0.05; ††*P*≤0.001.

Structural remodeling was investigated using micro-CT.^22^ In HF, LV free wall and chamber volumes were significantly increased, but right ventricular (RV) measurements were unaffected (Figure 2A and 2B). Representative 3-dimensional surface renderings of segmented free-running PFs from the LV of control and HF hearts are shown in Figure 2C through 2F and Figure I in the Data Supplement. HF PFs were hypertrophied and the network showed increased volume, free-running length, and longest free-running segment length (Figure 2G through 2I). PF free-running network length correlated strongly with LV chamber volume (Figure II and Table I in the Data Supplement).

### Gene Expression Changes in HF

**Figure 3.**
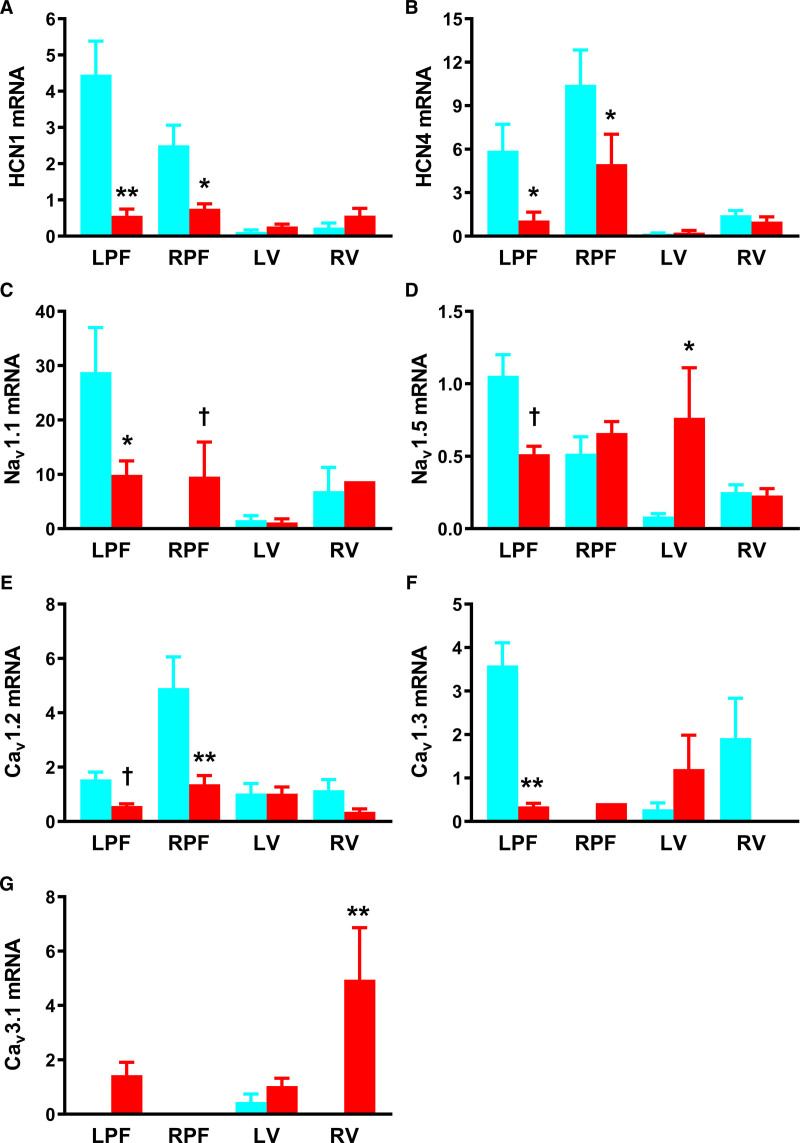
**Remodeling of transcripts for inward current-carrying ion channels in the Purkinje fibers (PFs) in heart failure (HF)**. **A–G**, mRNA abundance for various ion channels in the left PFs (LPFs) and right PFs (RPFs), left ventricle (LV) and right ventricle (RV) of control (blue bars), and HF (red bars) rabbit hearts. Data are presented as mean±SEMs; tissue samples per group=6–8; samples successfully amplified/detected per group, n=1–8. Ca_v_ indicates voltage-gated Ca^2+^; HCN, hyperpolarization-activated cyclic nucleotide-gated; and Na_v_, voltage-gated Na^+^. Statistical differences between control and HF rabbits were assessed using 2-way ANOVA (*) or *t* test (†); *,†*P*≤0.05; **P≤0.001.

Relative abundance of 40 transcripts for ion channels, Ca^2+^-handling proteins, gap junction subunits, proinflammatory, and fibrosis markers was measured in left and right PFs (LPFs and RPFs), LV, and RV muscle (Figures 3 through 5; Figure III and Table II through VI in the Data Supplement). Widespread changes were observed.

**Figure 4.**
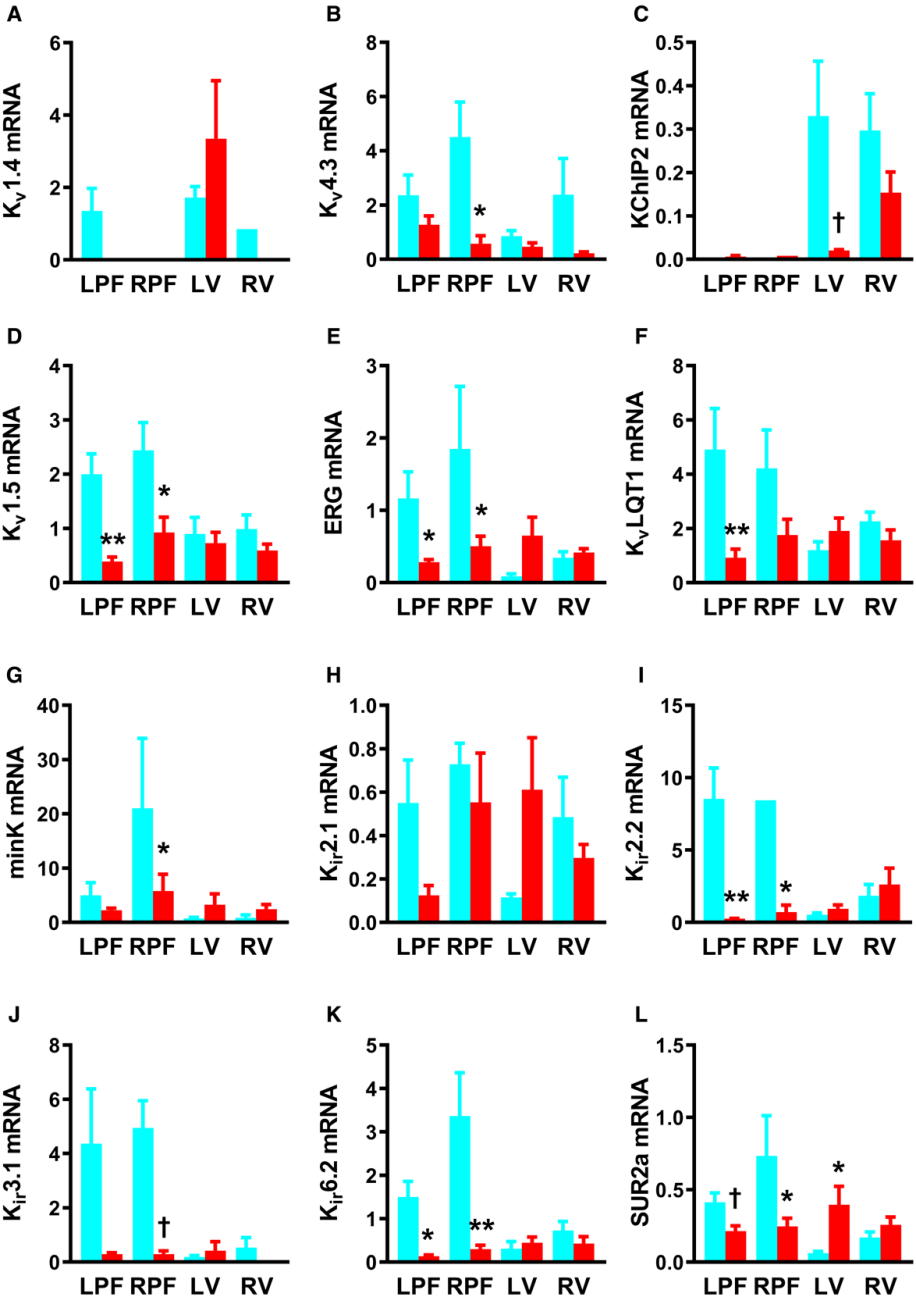
**Remodeling of transcripts for K^+^ channels and subunits in the Purkinje fibers (PFs) in heart failure (HF)**. **A–L**, mRNA abundance for various K^+^ channels in the left PFs (LPFs) and right PFs (RPFs), left ventricle (LV) and right ventricle (RV) of control (blue bars), and HF (red bars) rabbit hearts. Data are presented as mean±SEMs; tissue samples per group=6–8; samples successfully amplified/detected per group, n=1–8. ERG indicates Ether-à-go-go-related gene; KChIP, voltage-gated potassium channel-interacting protein; K_ir_, inward-rectifying potassium channel; K_v_, voltage-gated potassium channel; K_v_LQT, voltage-gated potassium channel subfamily Q; minK, minimal potassium channel subunit; and SUR, sulfonylurea receptor. Statistical differences between control and HF rabbits were assessed using 2-way ANOVA (*) or *t* test (†); *,†*P*≤0.05; **P≤0.001.

**Figure 5.**
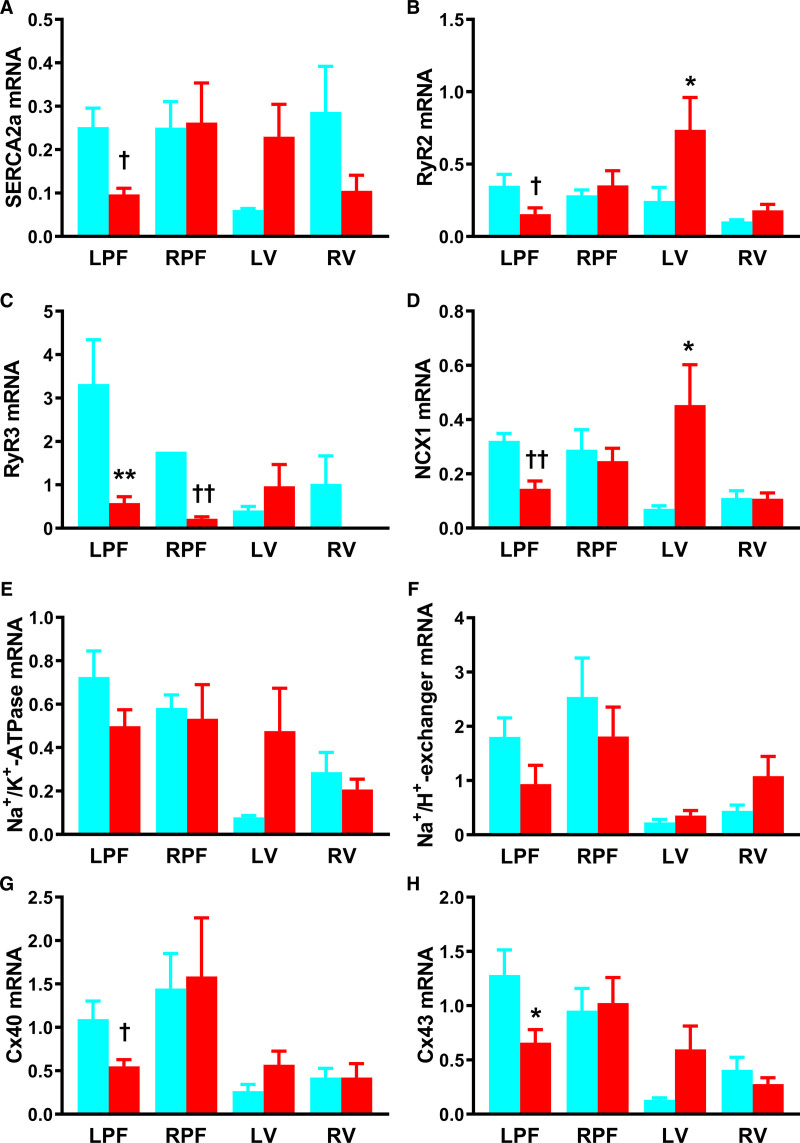
**Remodeling of transcripts for Ca^2+^- and Na^+^-handling proteins and connexins in the Purkinje fibers (PFs) in heart failure (HF)**. **A–H**, mRNA abundance for Ca^2+^- and Na^+^-handling proteins and Cx (connexins) in the left PFs (LPFs) and right PFs (RPFs), left ventricle (LV) and right ventricle (RV) of control (blue bars) and HF (red bars) rabbit hearts. Data are presented as mean±SEMs; tissue samples per group=6–8; samples successfully amplified/detected per group, n=2–8. NCX1 indicates Na^+^-Ca^2+^ exchanger 1; RyR, ryanodine receptor; and SERCA2a, sarco/endoplasmic reticulum Ca^2+^-ATPase. Statistical differences between control and HF rabbits were assessed using 2-way ANOVA (*) or *t* test (†); *, †*P*≤0.05; **, ††*P*≤0.001.

Downregulation of HCN isoforms and Na^+^- and Ca^2+^-channel subunits: HCN channels are responsible for *I*_f_, an important contributor to spontaneous phase-4 diastolic depolarization in PFs.^14^ HCN1 and HCN4 showed reduction in HF, in LPFs and RPFs (Figure 3A and 3B). The neuronal Na^+^-channel, Na_v_1.1 (voltage-gated Na^+^ channel 1.1), was reduced in LPFs but was unchanged in ventricle in HF. Na_v_1.1 was upregulated in RPFs on *t* testing (Figure 3C). Channel Na_v_1.5 (voltage-gated Na^+^ channel 1.5) is largely responsible for Na^+^ current (*I*_Na_) and mRNA for this channel was abundant in LPFs. Two-way ANOVA failed to show a difference in Na_v_1.5 expression in LPFs and RPFs in HF, but LPFs showed fall in channel expression on *t* testing. In HF, Na_v_1.5 was increased in the LV (Figure 3D). In dog PFs, Ca_v_1.2 (voltage-gated Ca^2+^ channel 1.2) is principally responsible for the L-type Ca^2+^ current (*I*_Ca,L_).^29^ Ca_v_1.2 mRNA was reduced in both LPFs and RPFs in HF (Figure 3E). Ca_v_1.3 (voltage-gated Ca^2+^ channel 1.3) also contributes to *I*_Ca,L_ in PFs,^29^ and Ca_v_1.3 expression was reduced by ≈90% in LPFs in HF (Figure 3F). We found no change in mRNA expression of either Ca_v_1 in ventricle in HF. Expression of Ca_v_3.1 (voltage-gated Ca^2+^ channel 3.1), which codes for the T-type Ca^2+^-channel, was unchanged in PFs and LV, but upregulated in RV (Figure 3G).

Expression of K^+^-channel subunits: subunits K_v_1.4 (voltage-gated K^+^ channel 1.4) and K_v_4.3 (voltage-gated K^+^ channel 4.3), contribute to the transient outward current (*I*_to_) in cardiac tissue. We observed no significant change in K_v_1.4, but K_v_4.3 was reduced in RPFs in HF (Figure 4A and 4B). The KChIP2 (K^+^ channel interacting protein 2), which regulates the repolarizing K_v_4.3 current and depolarizing Ca_v_1.2 current^30,31^ was downregulated in LV (*t* testing) but unaffected elsewhere in HF (Figure 4C). Delayed rectifier K^+^-channel subunits, K_v_1.5, ERG (Ether-à-go-go-Related Gene), K_v_LQT1 (voltage-gated K^+^ channel subfamily Q), and minK (KCNE1, minimal K^+^ channel subunit) are responsible for the ultrarapid outward current (*I*_K,ur_), rapid delayed rectifier current (*I*_K,r_) and slow delayed rectifier current (*I*_K,s_), respectively. In PFs K_v_1.5 and ERG were reduced in HF (Figure 4D and 4E). ERG was unaffected in ventricle. Expression of the slow delayed rectifier subunit K_v_LQT1 was reduced in LPFs, and minK was reduced in RPFs in HF (Figure 4F and 4G). These K^+^-channel subunits were unaffected in ventricle.

The inward rectifier K^+^-channel, K_ir_2.1 (inward-rectifier K^+^ channel 2.1), was unaffected in HF (Figure 4H); however, the apparently more abundant K_ir_2.2 (inward rectifier K^+^ channel 2.2) isoform was reduced in PFs (Figure 4I). No changes were seen in ventricle (Figure 4H and 4I). The acetylcholine-activated K^+^-channel subunit, K_ir_3.1 (acetylcholine-activated K^+^ channel 3.1) was not changed in LPFs but was reduced in RPFs on *t* testing (Figure 4J). K_ir_6.2 (ATP-sensitive K^+^ channel 6.2) and SUR2a (sulfonylurea receptor 2a) are responsible for the ATP-sensitive K^+^ current (*I*_K,ATP_) and in HF both transcripts were reduced in PFs (Figure 4K and 4L). SUR2a was upregulated in LV on *t* testing (Figure 4L).

Expression of Ca^2+^- and Na^+^-handling molecules: in HF, LPFs had reduced levels of mRNA for sarco/endoplasmic reticulum Ca^2+^- ATPase (SERCA2a) and ryanodine receptor (RyR2) (both on *t* testing) and RYR3, whereas in RPFs only RyR3 was downregulated (*t* testing; Figure 5A through 5C). In LV, RYR2 levels increased (Figure 5A and 5B). NCX1 (Na^+^-Ca^2+^ exchanger 1) was reduced in LPFs (*t* testing) and upregulated in LV (Figure 5D) in HF. Na^+^/K^+^-ATPase and Na^+^/H^+^-exchanger were unaffected (Figure 5E and 5F).

Connexins and expression of proinflammatory and fibrotic markers: Connexins are gap junction subunits facilitating cell-cell communication, both electrically and metabolically. In HF, levels of Cx40 (*t* testing) and Cx43 mRNA were reduced in LPFs, but not in RPFs or ventricles (Figure 5G and 5H). Few changes to thirteen proinflammatory and fibrosis-related transcripts were seen in HF (Figure III in the Data Supplement).

### Cellular Hypertrophy and Changes in Protein Expression in HF

**Figure 6.**
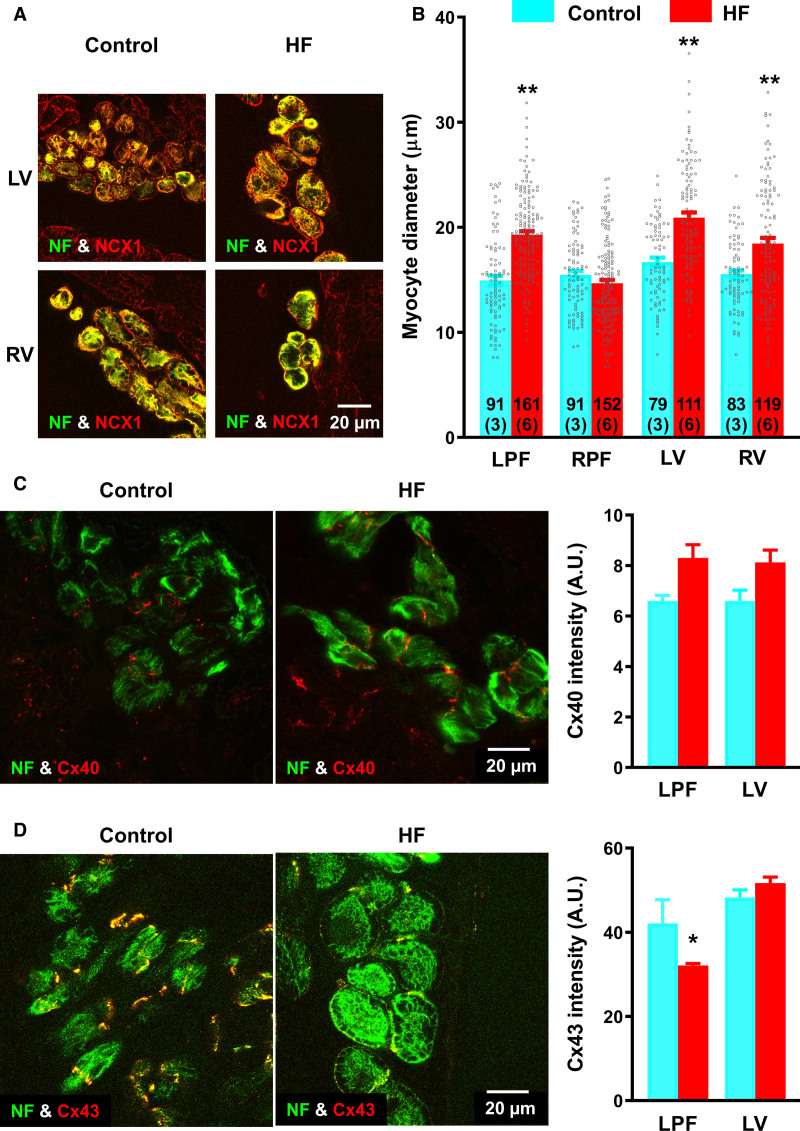
**Hypertrophic remodeling of myocytes and Cx (connexin) protein expression in heart failure (HF)**. **A**, Immunohistochemical double labeling of neurofilament (NF [neurofilament]; green signal) and NCX1 (Na^+^-Ca^2+^ exchanger 1; red signal) in left ventricular (LV; **top**) and right ventricular (RV; **bottom**) tissue sections of control and HF hearts. The tissue sections encompass Purkinje tissue (NF expressing) and ventricular muscle (NF nonexpressing). **B**, Regional changes in diameter of left Purkinje fiber (LPF), right Purkinje fiber (RPF), LV, and RV myocytes in control (3 hearts; blue bars), and HF (6 hearts; red bars) rabbits. Number of myocyte measurements for each group (n) is denoted within the bars and figures in brackets represent the number of hearts. **C**, **left**, Immunohistochemical double labeling of NF (green) and Cx40 (red) in LV of control and HF rabbits. **C**, **right**, Mean Cx40 fluorescence intensity measurements from LPFs and LV of control (n=3) and HF (n=5) rabbit hearts. **D**, **left**, Immunohistochemical double labeling of NF (green) and Cx43 (red) in LV of control and HF rabbits. **D**, **right**, Mean Cx43 fluorescence intensity measurements from LPFs and LV of control (n=3) and HF (n=5) rabbit hearts. Scale bar represents 20 µm. A.U. indicates arbitrary units. Data are presented as mean±SEMs. Statistical differences between control and HF rabbits were assessed using 2-way ANOVA. *P≤0.05; **P≤0.001.

Cardiac hypertrophy was demonstrated by increased heart/body weight ratio in this model (Figure 1B). Due to the spatial orientation of myocytes in PFs and ventricular walls, cell diameter is primarily responsible for changes in PF and ventricular wall thickness. Cell diameter was measured in confocal image scans of ventricular tissue sections double-labeled for neurofilament (PF specific marker^3^) and NCX1 (expressed in all cardiomyocytes; Figure 6A). In control sections, myocyte diameter was similar in LPFs and RPFs, LV, and RV (Figure 6B). In HF, diameter increased in LPFs (19.3±0.3 μm HF, 14.9±0.4 μm control), LV (20.9±0.5 μm HF, 16.7±0.4 μm control) and RV (18.5±0.5 μm HF, 15.5±0.4 μm control). RPFs were unaffected (Figure 6B). Myocyte diameter increase was more pronounced on the left side of the heart (LPFs and LV) compared with the right side (RPFs and RV) consistent with the insult to the left side in this HF model.

Observed QRS prolongation in HF is a result of slower conduction through PFs. Conduction slowing can occur through a reduction in myocyte-coupling because of changes in gap junction connexin expression. Using immunohistochemistry we investigated expression of 2 major Cx proteins, Cx40 and Cx43 (Figure 6C and 6D). Cx40 protein staining was not significantly altered in HF. However, Cx43 labeling was significantly decreased in LPFs in HF (Figure 6C and 6D), and this could contribute to slowed conduction.

LPFs were studied using volume electron microscopy, making use of Serial Block Face Scanning Electron Microscopy, allowing 3-dimensional-reconstruction of Purkinje myocytes, nuclei, and cell-cell junctions. In HF, fewer distinct cell-cell junctions were observed and the aggregated surface area of cell-cell junctions as a proportion of cell surface area was reduced (0.05±0.01 HF, 0.10±0.01 control; Figures IV and V in the Data Supplement).

### Action Potential Remodeling in HF

**Figure 7.**
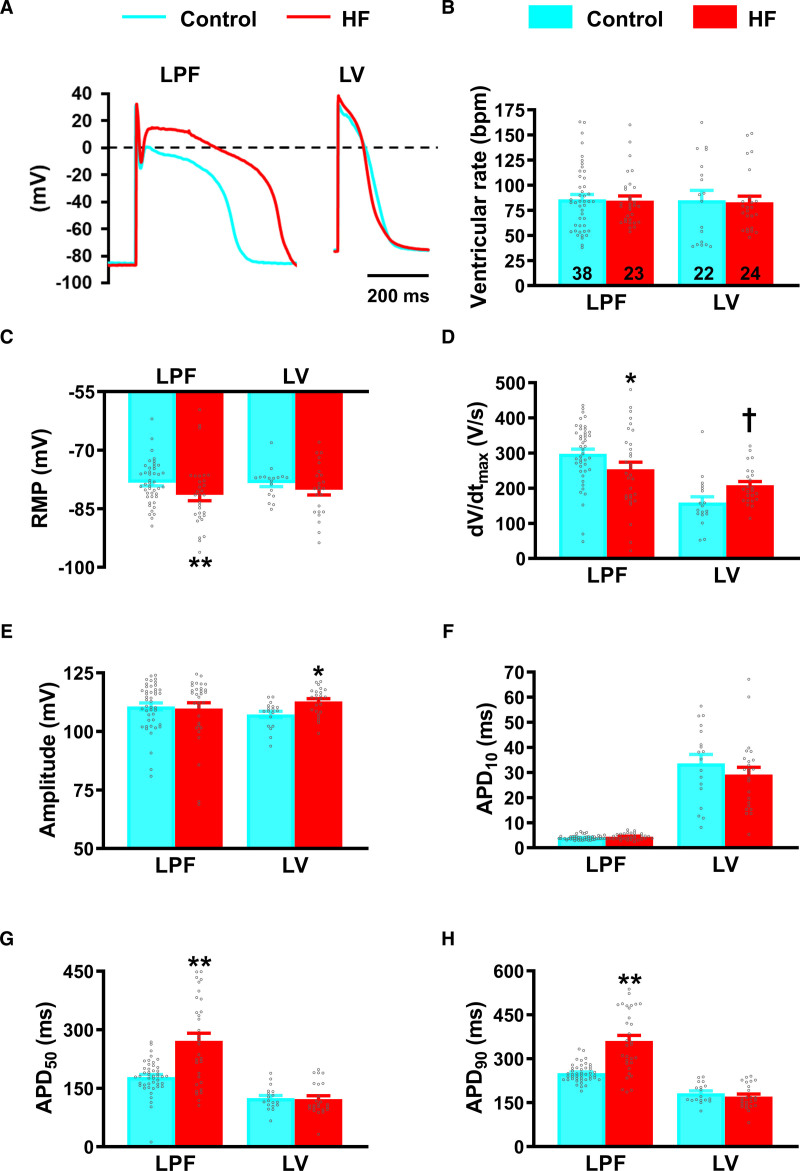
**Remodeling of the Purkinje fiber (PF) action potential in heart failure (HF)**. **A**, Typical records of free-running left PF (LPF) and left ventricular (LV) action potentials in control (blue) and HF (red) rabbit hearts. **B–H**, Measurements of ventricular beating rate (**B**), resting membrane potential (RMP; **C**) maximum upstroke velocity of the action potential (dV/dt_max_; **D**), action potential amplitude (**E**) and action potential duration (APD) at 10%, 50%, and 90% repolarization (**F–H**). Data are presented as mean±SEMs of 6 control and 6 HF hearts. Number of myocytes (ie, microelectrode impalements) for each group (n) are denoted within the bars in **B**. Statistical differences between control and HF rabbits were assessed using 2-way ANOVA (*) or *t* test (†); *,†*P*≤0.05; **,††*P*≤0.001.

The majority of gene expression changes in HF were observed on the left side of the heart, particularly in the LPFs, and therefore, we recorded intracellular action potentials from free-running LPFs and LV endocardium in superfused cardiac preparations with atria removed (Figure VI and Table VI in the Data Supplement). Heterogeneous changes in action potential morphology were observed in HF (Figure 7A). Spontaneous ventricular beating rate remained unchanged with HF (Figure 7B). Resting membrane potential was more negative in LPFs in HF, and unaffected in LV (Figure 7C). dV/dt_max_ was significantly reduced in LPFs but elevated in the LV in HF (Figure 7D). Action potential amplitude was higher in the LV in HF (Figure 7E). APD at 10% repolarization (APD_10_) was unaffected (Figure 7F). APD_50_ and APD_90_ increased almost 2-fold in LPFs in HF but remained unchanged in LV (Figure 7G and 7H).

**Figure 8.**
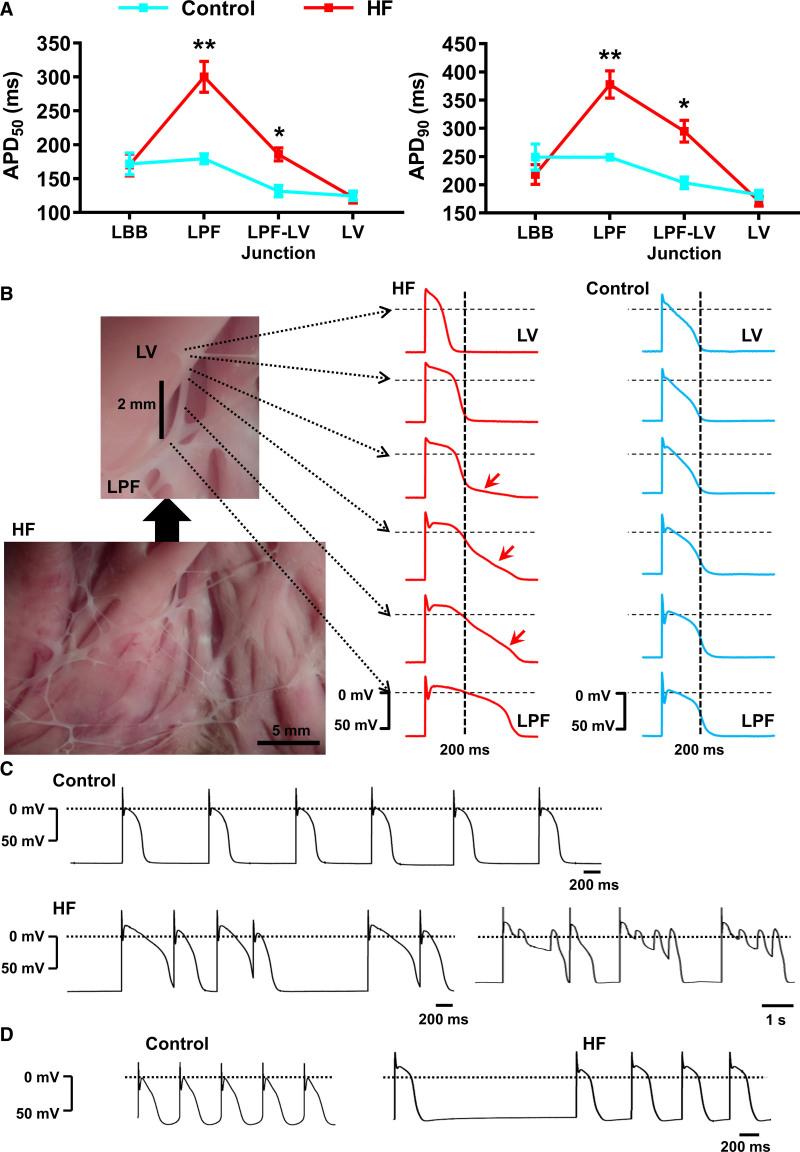
**Electrophysiological remodeling of the Purkinje fiber (PF)–ventricular junction in heart failure (HF)**. **A**, Action potential duration at 50% (APD_50_; **left**) and 90% (APD_90_; **right**) repolarization in the left bundle branch (LBB), left PF (LPF), LPF–left ventricular (LV) junction and LV of control (blue) and HF (red) hearts. Data are presented as mean±SEMs of control (6 hearts; 11 LBB, 38 LPF, 11 LPF-LV junction, and 18 LV myocytes/microelectrode impalements) and HF (6 hearts; 12 LBB, 23 LPF, 33 LPF-LV junction, and 24 LV myocytes). Statistical differences between control and HF rabbits were assessed using 2-way ANOVA followed by Sidak multiple comparison test. *P≤0.05; **P≤0.001. **B**, High (**top**) and low (**bottom**) magnification images of a typical LV preparation from a failing heart showing the free-running PFs. Typical action potential records from across a left PF-LV junction in the failing heart (from the sites shown by the arrows) are shown in red. For comparison, action potential records from a similar junctional region in a control heart are shown in blue. **C**, Representative LPF action potentials of a control heart (**top**) and examples of arrhythmogenic activity of LPFs (early afterdepolarizations and triggered action potentials) in a failing heart (**bottom**). **D**, Examples of pacemaker action potentials in LPFs of control (**left**) and failing (**right**) hearts. In **B–D**, the dashed lines correspond to 0 mV.

Our cardiac preparations allowed microelectrode access to the entire PF network. We recorded action potentials from the LBB and LPF-LV junction as well as the LPFs and LV (Figure 8A). Representative anatomic sites of recordings and typical action potential morphologies in control are shown in Figure VI in the Data Supplement. In control hearts, APD increased slightly from the LBB to free-running LPFs but was decreased at the LPF-LV junction; there was a further small decrease in LV (Figure 8A). At the LPF-LV junction, there was a gradual transition from PF action potential morphology with prominent early repolarization (phase-1) to the ventricular action potential with its characteristic morphology (Figure 8B). In HF, this picture was radically altered (Figure 8B). In HF, APD was unchanged in the LBB and LV, but in LPFs and at the LPF-LV junction, it almost doubled (Figure 8A; Figure VII in the Data Supplement); consequently, there were large APD gradients from the LBB to LPFs and from the LPFs to LV (Figure 8A). In HF, the large APD gradient at the LPF-LV junction, and electronic interaction over the space constant of the tissue (λ≈2 mm), resulted in bizarre abnormal action potential morphologies (Figure 8B).

In HF, LPFs showed with early afterdepolarizations and triggered activity (Figure 8C and 8D). Escape pacemakers with characteristic diastolic depolarization were observed in 40% of control and 66% of HF preparations. In control LPFs, pacemaker action potentials were rhythmic with constant cycle length, whereas in HF LPFs, pacemaker activity was aberrant and occurred in bursts with irregular cycle length (Figure 8D). Diastolic depolarization was absent at the LPF-LV junction and in LV.

## Discussion

The PF network has been linked to ventricular arrhythmias in structurally abnormal and diseased hearts.^7,32,33^ We have investigated the electrophysiological properties of PFs and ventricular myocardium in a rabbit model of congestive HF which features an enlarged dilated heart, reduced ejection fraction, evidence of conduction system dysfunction, and prolonged corrected QT interval, all clinical features of HF patients.^34–37^ In the rabbit model, in PFs there was hypertrophy, conduction slowing (evidenced by QRS prolongation, again characteristic of HF patients^4^), slowing of action potential dV/dt_max_, marked prolongation of APD, increased arrhythmic activity, and marked changes in expression of ion channels underlying the action potential. The data support a role for PFs in ventricular dysfunction and in arrhythmogenesis. Surprisingly, few changes were seen in LV—most research on the failing heart has focused on ventricular muscle and the role of PFs has been relatively neglected.

### Action Potential and Ion Channel Remodeling

A striking finding in this study is the discordance between the extent of the changes in LPFs compared with LV (Table II through VI in the Data Supplement). mRNA levels for 40 ion channels, Ca^2+^-handling molecules, connexins, and proinflammatory and fibrosis markers were assessed: in HF, 50% and 35% of these were dysregulated in LPFs and RPFs, respectively, whereas in contrast, only 12.5% and 7.5% were dysregulated in LV and RV, respectively. There was a marked reduction in expression of HCN channels, Na^+^-channels, Ca^2+^-channels, K^+^-channels, Ca^2+^-handling molecules, and connexins in LPFs in HF (Figures 3 through 6). Prolongation of the LPF action potential (Figure 7) could be the result of the downregulation of the important K^+^-channels, K_v_1.5, ERG, and K_v_LQT1 (Figure 4), which are responsible for *I*_K,ur_, *I*_K,r_, and *I*_K,s_, respectively, and are known to control APD. Downregulation of Na^+^-channels (Figure 3) could account for the slowing of dV/dt_max_ of the LPF action potential in HF (Figure 7) which, together with downregulation of Cx43 (Figures 5 and 6), would lead to impaired conduction and account for the observed QRS prolongation (Figure 1). In a model of tachypacing-induced HF Maguy et al^38^ observed conduction slowing in LPFs, and they suggested that consequent dyssynchronous ventricular activation (and consequently contraction) could give rise to adverse remodeling in HF. Our data provide strong support for this viewpoint, and the disparity between the changes in LPFs and LV may indicate a more prominent role for PF remodeling in the volume-induced and pressure-overload–induced rabbit HF model.

The marked LPF changes led us to use micro-CT imaging, which has demonstrated for the first-time massive hypertrophy and increased length of the whole free-running LPF system in HF. The initial and principal lesion in this model is severe acute aortic regurgitation, which we propose may cause PF remodeling through LV dilatation and stretch of the PF network. Such mechanisms may also be operative in the peri-infarct zone following myocardial infarction (Purkinje system is preserved in the subendocardium in myocardial infarction^39^), in acute valvular lesions following endocarditis or leaflet avulsion, and in viral and neonatal dilated cardiomyopathy.

It is possible that downregulation of ion channels and Cx43 etc in the LPFs in HF is a result of physical trauma as a consequence of the stretch of the PF network. Serial Block Face Scanning Electron Microscopy investigations revealed signs of cell damage—nuclear membrane dissolution and intranuclear mitochondria in LPF myocytes in HF (Figure V in the Data Supplement).

This is the first study in which RPF gene expression has been examined in HF. We found downregulation of HCN but not Na^+^-channels, one of the Ca^2+^-channels and various K^+^-channels, little change in Ca^2+^-handling molecules, and no change in connexins (Figures 3 through 5). Less severe changes in the action potential of RPFs are therefore expected.

Most electrophysiological studies in HF have focused on LV myocardium, but here we observed few changes in the expression of ion channels, Ca^2+^-handling proteins, connexins, and proinflammatory and fibrosis markers in LV. There was increased expression of the main Na^+^-channel, Na_v_1.5 (Figure 3), which could be responsible for the elevated dV/dt_max_ and amplitude of the LV action potential (Figure 7). Therefore, there is evidence of increased active (but not passive—connexins unchanged in LV) determinants of conduction velocity in LV. There was no change in APD in HF and consistently, almost no change in K^+^-channel expression in LV (Figure 4). Two key Ca^2+^-handling molecules, RyR2 and NCX1, were upregulated. NCX1 upregulation and SERCA2 downregulation are common findings in HF,^40^ although we found no change in SERCA2 in LV in this model.

### PF Arrhythmogenesis

In the present study, arrhythmic changes were evident in HF PFs. Early afterdepolarizations and triggered activity were frequent (Figure 8). Early afterdepolarizations can be attributed to the APD prolongation (Figure 8) presumably because of downregulation of repolarizing K^+^ currents (Figure 4). Occurrence of triggered activity is surprising because HCN1 and HCN4 pacemaker channels were downregulated in HF PFs (Figure 3). However, inward rectifier K^+^-channels (K_ir_2.2, K_ir_3.1, and K_ir_6.2) especially K_ir_2.2 (the most abundant of the channels) were downregulated (Figure 4) and this is expected to promote triggered activity.

PF arrhythmias are well known to result from dysfunction of intracellular Ca^2+^-handling.^41^ In the present study 6 key Ca^2+^-handling transcripts were reduced, Ca_v_1.2, Ca_v_1.3, SERCA2a, RyR2, RyR3, and NCX1, in the LPFs in HF (Figures 3 and 5). There is known to be a maladaptive remodeling of intracellular Ca^2+^-handling in ventricles of failing hearts, and downregulation of SERCA2a is common.^40^ However, there can be NCX1 upregulation in failing ventricles,^40^ whereas in the LPFs of failing hearts the opposite was observed (Figure 5). If transcript changes in the LPFs translate into protein changes, profound functional changes are anticipated, but implications for arrhythmogenesis can only be speculated on. Interestingly, RyR3 was more abundant than the cardiac ryanodine receptor, RyR2. RyR3 has previously been reported to be important in PFs.^42^

### The Gate Hypothesis

In the dog, Myerberg et al^43,44^ observed that APD increases progressively from the His-bundle and reaches a maximum of 2 to 3 mm proximal to the termination of PFs and distal to this APD shortens progressively. A qualitatively similar change in APD was observed here in control rabbit hearts, although increases in APD from the LBB to LPFs were modest (Figure 8). However, this pattern was much more marked in failing hearts (Figure 8). Because APD normally determines the refractory period, the area of maximum APD in the Purkinje network has been referred to as a gate, and this gate determines the minimum possible coupling interval between a regular action potential and a premature one, for the premature action potential to be conducted to ventricular muscle.^43,44^ The gate, therefore, protects against hazardous prematurity.^43^ Reentry based on the gate hypothesis has been proposed,^45^ in which a premature action potential may be conducted across the gate in one branch of the Purkinje network but not another (naturally there are differences in refractoriness in different branches^45^) and after conducting through ventricular muscle may then be able to retrogradely propagate through the gate previously refractory. Such reentry will also be dependent on slow conduction in the reentry loop; it will be facilitated by the well-known conduction delay of 5 to 20 ms at the Purkinje-ventricle junction.^46,47^ This type of reentry may be more likely in the failing heart, because the changes in APD (and, therefore, refractoriness) are more marked and there is a slowing of PF conduction. In HF, because of the prolonged APD in LPFs, but not in LV, at the LPF-LV junction there was a marked APD gradient over a short distance. This raises the possibility that prolonged depolarizations of distal LPFs could re-excite LV and generate ectopic beats.

In the clinical setting, congestive HF often results from systolic LV dysfunction and development of secondary mitral regurgitation. In this context, our rabbit model is of much relevance. LBBB is common in systolic HF patients^4^ and such patients may get cardiac resynchronization therapy. The LPF remodeling in acute HF presented here occurs before long-term LBBB. We may speculate that the APD prolongation (and consequently refractory period), if left to deteriorate, can result in LBBB. Biventricular pacing has proven effective in HF patients with severely reduced LV ejection fraction and LBBB.^48^ Our data would suggest that His-bundle pacing is less likely to help during the development of acute, severe Purkinje network dysfunction. We cannot exclude a possible benefit of His-bundle pacing once LV dilatation has become chronic and PF remodeling has become permanent.^49^ The clinical goal should be to optimize cardiac hemodynamics by taking every measure available to reduce the *development* of LV dilatation associated with volume-overload.^50^

### Conclusions

In HF, there is a widespread remodeling of Purkinje network at the structural, molecular, and electrical levels resulting in dysfunction and an arrhythmic substrate. Treatment strategies that forestall LV dilatation may prevent PF remodeling and are likely to benefit HF patients.

## Acknowledgments

We are grateful to the staff of the Henry Moseley X-ray Imaging Facility for help with the micro–computed tomography (CT) scanning made available through Engineering and Physical Sciences Research Council (EPSRC) grant funding EP/M010619/1 and EP/M022498/1. The authors wish to thank the staff in the Faculty of Biology Medicine and Health Electron Microscopy Core Facility for their assistance and the Wellcome Trust for equipment grant support to the Electron Microscopy Facility.

## Sources of Funding

Funding for this work was provided by British Heart Foundation programme grants (RG/11/18/29257 and PG/15/16/31330) with support to Christine Stalker and Kate Dutton for provision of animal care at the University of Liverpool, a Fondation Leducq grant (TNE FANTASY 19CV03), and by a grant from the Cardiac Surgical Research Fund of the Alder Hey Children’s National Health Service Foundation Trust. Dr Nakao is supported by funding from the Naito Foundation and the Nakatomi Foundation, Japan.

## Disclosures

None.

## Supplemental Materials

Supplemental Methods

Supplemental Results

Supplemental Discussion

References 51–54

Author Contributions

Figures I–VII

Tables I–VII

## Supplementary Material


