# Supplementary material for: Remodeling of the Purkinje Network in Congestive Heart Failure in the Rabbit
Source: Circ Heart Fail. 2021 Jul 12;14(7):e007505. doi: 10.1161/CIRCHEARTFAILURE.120.007505 (PMC8288482; doi:10.1161/CIRCHEARTFAILURE.120.007505)
Supplement: Supplementary file 1 [file hhf-14-e007505-s001.pdf]

SUPPLEMENTAL MATERIAL

Remodelling of the Purkinje network in congestive heart failure in the rabbit

Sunil Jit R.J. Logantha, Xue J. Cai, Joseph Yanni, Caroline B. Jones, Robert S. Stephenson, Luke Stuart, Gillian Quigley, Oliver Monfredi, Shu Nakao, Il-Young Oh, Tobias Starborg, Ashraf Kitmitto, Akbar Vohra, Robert C. Hutcheon, Antonio F. Corno, Jonathan C. Jarvis, Halina Dobrzynski, Mark R. Boyett, George Hart

Supplemental Methods

Echocardiography and ECG

Rabbits were held in a supine or lateral decubitus position and precordial fur was shaved. 2D images were obtained in the parasternal long axis view and colour Doppler was used to assess the aortic and mitral valves for regurgitation. For measurement of ventricular dimensions, the M-mode cursor was placed at the tip of the mitral leaflets, perpendicular with the interventricular septum. Left ventricular fractional shortening was calculated using the Teichholz formula.

For ECG recording, needle electrodes were inserted subcutaneously in the right and left forelimbs and left hind limb. The signal was sampled at 1 kHz, amplified, filtered (Neurolog, Digitimer, UK) and passed through an analog-to-digital converter (PowerLab, AD Instruments, Australia), before being recorded on disk using LabChart Pro Software (AD Instruments, Australia). Once the ECG signal was stable, ~2000 consecutive beats were recorded as baseline, prior to autonomic blockade using intravenous atropine and propranolol. Atropine 1 mg was given first then propranolol, 3 mg over 5 min, followed by a further 1 mg atropine. ECG recording continued for ~2000 consecutive beats, and the animal was killed by a Schedule 1 method. The ECG signals obtained were interrogated offline. RR, PR, QRS, QT and QTc intervals were derived from appropriate 500 beat runs of stationary ECG data in a semi-automated way using the LabChart Pro software suite.

Micro-CT imaging and data handling

Perfusion fixed hearts were removed from phosphate buffered formal saline (PBFS), drained and blotted dry. Hearts were then fully immersed in I_2KI contrast agent (iodine crystals (I_2), potassium iodide (KI), added to PBFS) at 3.75% or 7.5%, for 3-5 days. All Hearts were micro-CT imaged with the Nikon Metris XTEK 320 kV Custom Bay system at the Henry Moseley X-Ray Imaging Facility, University of Manchester. Imaging parameters were optimised for each specimen to maximise spatial and contrast resolution and to facilitate data handling. Scans were acquired using a copper or molybdenum target with X-ray energies ranging from 90-160 kV. 1440-3500 projections within the 360° rotation were recorded on a 2K x 2K Perkin Elmer 1621-16-bit amorphous silicon flat-panel detector with 200 µm pixel pitch, resulting in scan times of approximately 20-50 min. All scans were reconstructed using Nikon Metrolasis CT-Pro software (Metris XT 1.6). Isometric resolutions for whole scans ranged from 19-29 µm.

The free-running Purkinje networks were segmented using a self-devised, semi-automatic segmentation technique. This approach used two stages: a primary segmentation based on inclusion of the region of interest (ROI) with its immediately surrounding air volume (e.g. black voxels), and a secondary segmentation based on differential attenuation. The ROIs were initially selected using an oversized 'brush' tool (Amira 5.4.0) to incorporate both the ROI and a thin shell of empty space. This allowed for interpolations between large numbers of slices (10-200), making this a highly efficient method. The selection was then stored, and the range of voxel values within the anatomically defined ROI were recorded. Using the 'threshold' tool (Amira 5.4.0) a masking window was then applied to the stored selection that identified, and thus discriminated, the ROI from the surrounding tissue or empty space. Segmented ROIs were then smoothed and any small islands were removed. Tissue volumes were calculated in Amira 5.4.0.

The 3D filament analysis (Amira 5.4.0) was used to investigate and quantify the free-running Purkinje fibre network. Originally developed for use in analysis of nerve dendrites, the *Auto Skeleton* tool (Amira 5.4.0) traced connected regions according to a user-defined window level and converted the

centreline of those regions into 3D skeletonised networks composed of points, segments, nodes, and loops. From these data, total free-running lengths and mean segment lengths were calculated.

qPCR

Total RNA was isolated from frozen samples using a modified Qiagen fibrous tissue protocol with a DNAse digestion step. The RNA was ethanol-precipitated, and the pellets were dissolved in 20 μl of RNAse-free water for reverse transcription with Superscript III reverse transcriptase (Invitrogen, U.S.A.).

The sequences for genes of interest were found using the search engines on Pubmed (www.ncbi.nlm.nih.gov/pubmed) and Ensembl (www.ensembl.org) websites. Primers were designed for the chosen sequences using Primer3 Input software (version 0.4.0) and tested using Netprimer (www.premierbiosoft.com) and BLAST (http://blast.ncbi.nlm.nih.gov) search tools. The primer sequence was tested and checked for folding using Mfold software (http://mfold.rna.albany.edu), and if satisfactory, the primers were manufactured by Eurofins (www.eurofinsdna.com). Primers were used in test qPCR reactions at three concentrations: 50, 300 and 900 nM, and the products were run on DNA gels. Only primers deemed acceptable, including neat amplification plots and absence of primer dimers, were used for further analysis. We have previously reported left and right primer sequences, along with corresponding reference sequences for the ion channels, Ca^{2+} handling molecules and connexins.[26] Supplemental Table VII summarises the successful primer sequences relating to pro-inflammatory and fibrosis transcripts.

Experiments were carried out to establish the most appropriate reference gene for the samples and primers for 28S and GAPDH genes were tested. The qPCR results were analysed by StatMiner software and stability of the two reference genes compared. 28S had a lower stability score than GAPDH and the combination of 28S and GAPDH was more stable. Each sample was run in triplicate.

Immunohistochemistry

Cryosections were fixed with a 1×Phosphate Buffer Saline (PBS) solution containing 4% paraformaldehyde (PFA, pH 7.3), blocked and permeabilised with 1×PBS solution containing 2%-normal donkey serum (NDS) and 0.5%-Triton X-100. Primary antibodies raised in mouse (anti-Cx40, anti-Cx43, anti-neurofilament, anti-NCX1; Chemicon, U.S.A.) were diluted 1/100 in 1×PBS solution containing 2%-NDS and 0.1%-Triton X-100 for overnight incubation with cryosections. FITC Fluor-conjugated donkey anti-mouse antibody (488-nm; Invitrogen, U.S.A.) or Cy3-conjugated donkey anti-mouse antibody (555-nm; Invitrogen, U.S.A.) were used as secondary antibodies (1/600 dilution). Slides were mounted in Vectashield mounting medium (Vector laboratories, UK) and examined with a Zeiss Axiovert 100-M microscope coupled to a Zeiss LSM-510 laser-scanning confocal system. Identical settings were used to image samples from control and congestive heart failure rabbits.

For cell diameter measurements 3 control and 6 HF hearts were used. Each heart was cryo-sectioned and ~10 sections from different levels were double immunolabelled for cell membrane proteins, NCX1 and neurofilament (positive marker for Purkinje cells). Immunofluorescence images were analysed using ImageJ (NIH). The majority of cells were assumed to be cut transversely, in the plane of their minimum dimension. The minimum diameter of the cell outline was measured, to compensate for any obliquity of sectioning. Cell diameters were averaged for 10-15 cells/image.

3D Serial Block Face Scanning Electron Microscopy

Freshly dissected tissue samples of free-running Purkinje fibres (1-2 mm length) were prepared for serial block face scanning electron microscopy (SBF-SEM) following established protocols for fixation, staining, dehydration, and resin embedding described previously.[51] Survey images of Purkinje fibres were initially collected using the FEI Quanta 250 FEG SEM (FEI Company) in order to assess the grade of staining and identify regions of interest with >5 Purkinje myocytes. The surveyed blocks were trimmed and coated with gold-palladium using a Quorum SC7620 sputter coater (Quorum Technologies). Tissue blocks were returned to the Quanta FEI 250 FEG equipped with a Gatan 3View ultramicrotome. Serial 200 nm sections were cut *in situ* with the inbuilt diamond knife and after each cut, the newly exposed block face was imaged with voxel size of 20 nm in the x-γ plane, and the process repeated until 1000 images were collected. Imaging conditions were 3.8

kV 0.5 Torr, 3.5 µs dwell time. 5000x5000 pixel images were collected at each slice, with periodic re-centering in order to keep the Purkinje fibre in the field of view. The data stacks were re-aligned prior to image analysis.

Image analysis was performed using IMOD software (University of Colorado Boulder). After examining image stacks for gross ultrastructural features, representative Purkinje myocytes and their ultrastructural features of interest (nuclei and cell-cell junctions) were annotated/segmented by manual contrast-based segmentation techniques.[52] Manual segmentation involved delineating boundaries of structures with a closed contour on every fifth image for myocytes and nuclei, and every consecutive image for cell-cell junctions, to produce an accurate 3D model for making morphometric measurements. IMOD allowed precise measurement of surface area of myocytes, nuclei and cell-cell junctions. In the case of nuclei with discontinuous membrane the contours were drawn to be continuous by connecting either ends to enable 3D reconstruction and morphometric measurements.

Supplemental Results

Expression of pro-inflammatory and fibrotic markers

Thirteen pro-inflammatory and fibrosis-related transcripts were measured, and few changed in HF. Results are displayed as changes in gene expression compared to control expression in Supplemental Fig. III. In the left Purkinje fibres, the only significant differences were a reduction in angiotensin II type-1 receptor and tissue inhibitor of metalloproteinase-4 (Supplemental Fig. IIIA). Extracellular matrix component fibronectin 1 was elevated in the right Purkinje fibres (Supplemental Fig. IIIB). No significant changes were seen in the left ventricle (Supplemental Fig. IIIC). Fibronectin 1 and another tissue inhibitor of metalloproteinases were elevated in the right ventricle in heart failure (Supplemental Fig. IIID).

Electron microscopy

Serial imaging of a transversely cut control heart free-running Purkinje fibre revealed the spatial organization of individual Purkinje myocytes surrounded by abundant collagen fibres and an outer layer of loose connective tissue (Supplemental Fig. IVA). 3D reconstructions revealed a central core of longitudinally running Purkinje myocytes running parallel to the direction of the fibre (Supplemental Fig. IVB). Purkinje myocytes were mononucleated and spindle shaped with a rough membrane with small surface projections (Supplemental Fig. IVC). Cell-cell junctions were identified as broad areas with closely opposed/fused sarcolemma (Supplemental Fig. IVC, left and inset) and tracked through the serial images. In control Purkinje myocyte, numerous cell-cell junctions were uniformly distributed throughout the sarcolemma providing an abundance of pathways for action potential propagation. In heart failure Purkinje myocyte, fewer cell-cell junctions were observed (Supplemental Fig. IVC,D). The ratio of cell-cell junction surface area to total cell surface area was reduced in heart failure (0.045±0.014 *vs.* 0.099±0.014 in control; n=3 myocytes, $P = 0.05$; Supplemental Fig. IVD,E). Additionally, in two out of three 3D reconstructed heart failure Purkinje myocytes, evidence of pathological remodelling suggestive of cell damage was observed: selective nuclear membrane discontinuity and mitochondrial entry (Supplemental Fig. V).

Supplemental Discussion

Action potential and ion channel remodelling-comparison to other studies

In a dog model of HF induced by rapid ventricular pacing,[38,53,54] phase-1 repolarization of the Purkinje fibre action potential was reduced and the plateau voltage was increased, but APD was unaffected.[54] There was a decrease in I_{to}, and background inward rectifier K^+ current, $I_{K,1}$, and a slowing of inactivation of $I_{Ca,L}$; the decrease in I_{to} can explain the decrease in phase-1 repolarization. The effects on the Purkinje fibre action potential in the dog HF model were, therefore, different from the effects on the Purkinje fibre action potential in the rabbit model in the present study in which there was no

obvious change in phase-1 repolarization, but a marked APD prolongation (Figs. 7 and 8); also in the rabbit model there were no significant changes in ion channel subunits responsible for I_{to} (K_v1.4, K_v4.3 and KChIP2) except for K_v4.3 in the RV (Fig. 4). Differences between the two studies may be species related, but could also be the result of different insults used to induce HF. The rapid ventricular pacing HF model has a clinical counterpart in tachycardia-induced ventricular cardiomyopathies. However, congestive HF is most often due to systolic LV dysfunction and development of secondary mitral regurgitation. In this context, the rabbit model of volume- and pressure-overload induced congestive HF is of much relevance.

Supplemental References

51. Linscheid N, Logantha S, Poulsen PC, Zhang S, Schrolkamp M, Egerod KL, Thompson JJ, Kitmitto A, Galli G, Humphries MJ et al. Quantitative proteomics and single-nucleus transcriptomics of the sinus node elucidates the foundation of cardiac pacemaking. **Nat Commun.** 2019;10:2889. DOI: 10.1038/s41467-019-10709-9

52. Kremer JR, Mastronarde DN, McIntosh JR. Computer visualization of three-dimensional image data using IMOD. **J Struct Biol.** 1996;116:71-76. DOI: 10.1006/jsbi.1996.0013

53. Li D, Melnyk P, Feng J, Wang Z, Petrecca K, Shrier A, Nattel S. Effects of experimental heart failure on atrial cellular and ionic electrophysiology. **Circulation.** 2000;101:2631-2638. DOI: 10.1161/01.cir.101.22.2631

54. Han W, Chartier D, Li D, Nattel S. Ionic remodeling of cardiac Purkinje cells by congestive heart failure. **Circulation.** 2001;104:2095-2100. DOI: 10.1161/hc4201.097134

Author Contributions

- Sunil Jit R.J. Logantha: helped to generate the rabbit model of heart failure, did all sharp microelectrode recordings, performed immunohistochemistry, assisted with electron microscopy analysis, carried out final data analysis, helped to write the paper, generated figures and revised the paper.
- Xue J. Cai: helped to generate the rabbit model of heart failure, to do the surgical procedures, to carry out qPCR, analyse data, generate figures, and write the paper.
- Joseph Yanni: helped to generate the rabbit model of heart failure, to dissect tissue samples for qPCR, to carry out the qPCR experiments, to generate rabbit qPCR primers, to do statistical analysis of data, to generate figures, to write the paper.
- Caroline B. Jones: carried out the echocardiography.
- Robert S. Stephenson: Performed micro-CT experiments including, *in situ* perfusion fixation of hearts, sample preparation, scanning, data analysis, wrote up relevant results and compiled figures.
- Luke Stuart: Performed electron microscopy image analysis.
- Gillian Quigley: carried out the qPCR measurements and analysis of inflammatory markers.
- Oliver Monfredi: assisted in the electrocardiogram measurements from the rabbit.
- Shu Nakao: Sample preparation and imaging with electron microscopy.
- Il Young Oh: Electron microscopy image analysis.
- Tobias Starborg: Electron microscopy sample preparation and imaging.
- Ashraf Kitmitto: Helped design and supervise electron microscopy imaging and analysis.
- Akbar Vohra: helped to generate the rabbit model of heart failure.
- Robert C. Hutcheon: helped to generate the rabbit model of heart failure.
- Antonio F. Corno: helped to generate the rabbit model of heart failure.
- Jonathan C. Jarvis: supervised the analysis of the micro-CT data.

- Halina Dobrzynski: raised funding for the work, contributed to overall supervision and design of immunohistochemistry experiments.
- Mark R. Boyett: helped initiate the project, raised funding for the work, helped design and supervise all aspects of the project, and helped write and revise the paper.
- George Hart: helped initiate the project, designed, and implemented the strategy for the development of a rabbit model of heart failure, participated in all aspects of the work on the rabbit model, helped supervise the project in general, wrote the first draft of the paper and revised the paper.

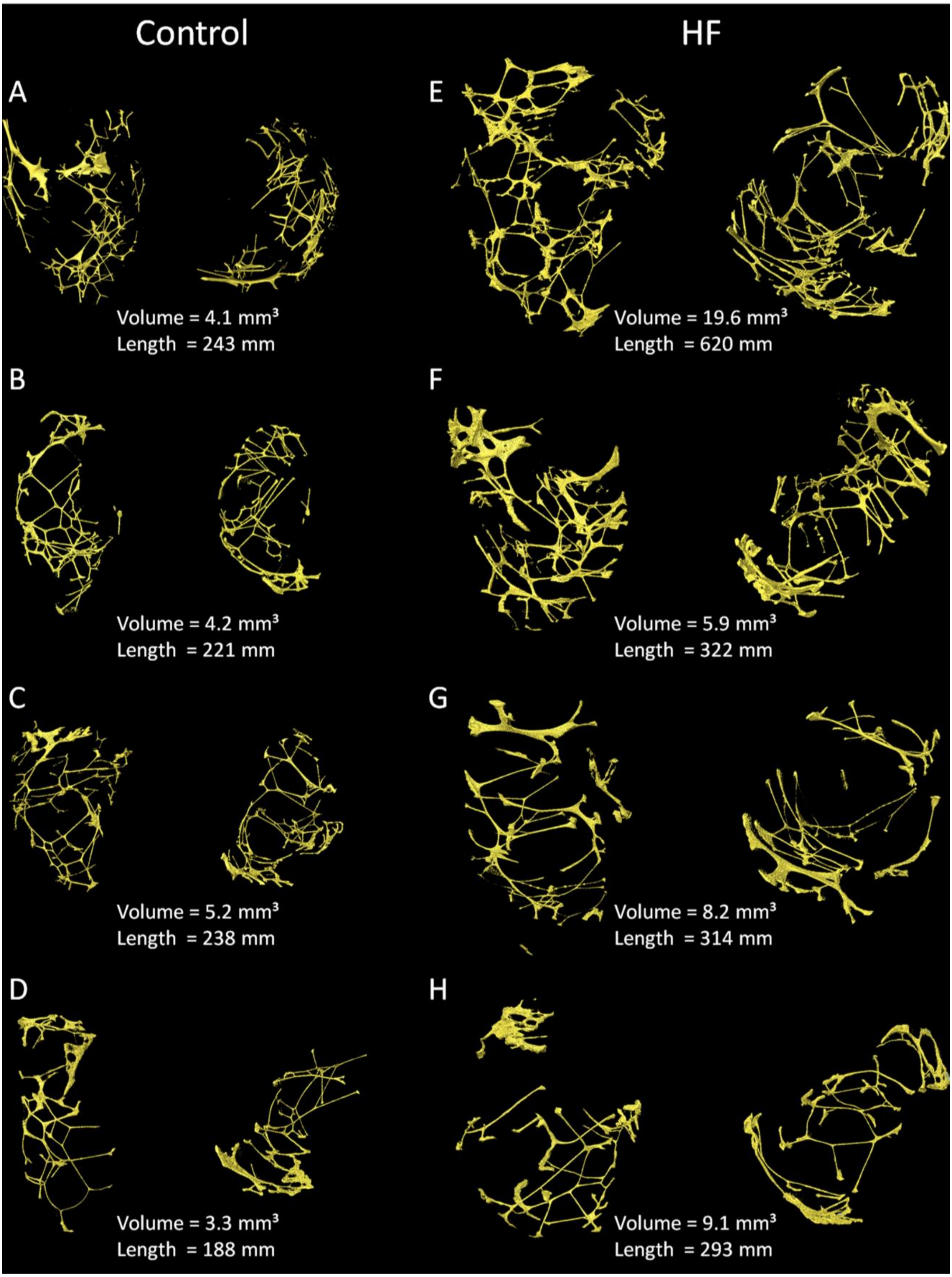

Supplemental Fig. I. 3D visualisation of the left ventricular free-running Purkinje network in control and failing hearts. A-H, scaled 3D surface renderings segmented from micro-CT data of control (A-D) and failing (E-H) hearts. For each heart, anterior (left) and superior (right) views are shown, and tissue volumes and total network lengths given.

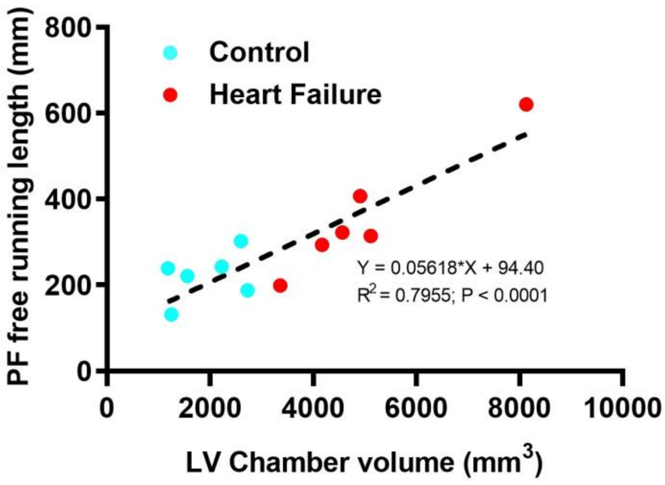

Supplemental Fig. II. Correlation plot between Purkinje fibre free running network length and the left ventricular chamber volume. Purkinje fibre (PF) free running network length is plotted against corresponding left ventricular (LV) chamber volume in control (blue) and failing (red) hearts. Measurements are from 6 control and 6 failing hearts. Best-fit trend line was fitted by linear regression and results are shown in inset.

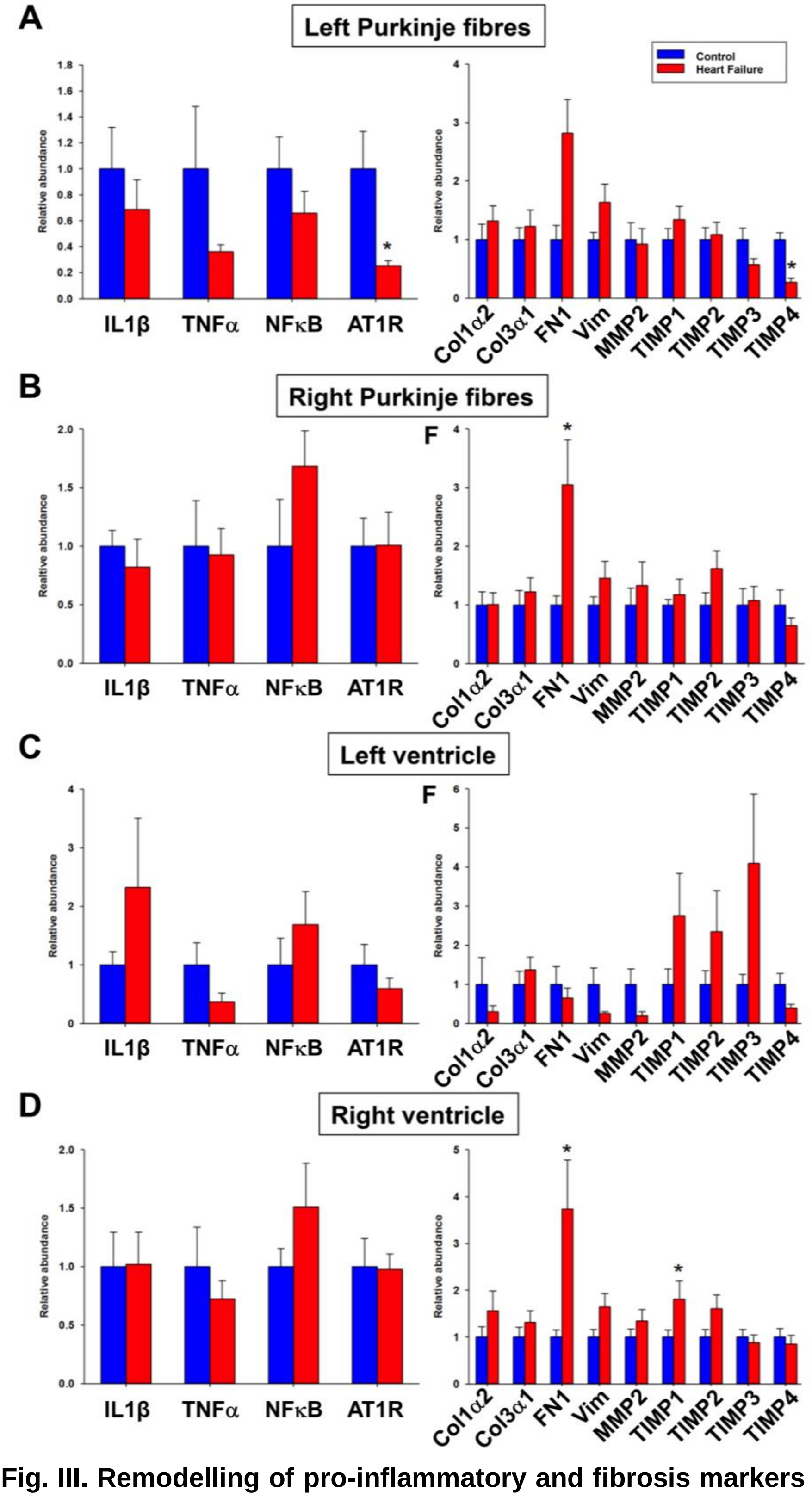

Supplemental Fig. III. Remodelling of pro-inflammatory and fibrosis markers in the Purkinje fibres in heart failure. Abundance of pro-inflammatory and fibrosis transcripts is shown in the left Purkinje fibres (A), right Purkinje fibres (B), left ventricle (C) and right ventricle (D) of control (blue) and failing (red) hearts. Data are presented as mean±SEM (control, n=5-8; heart failure, n=6-7). **P*≤0.05 *versus* corresponding control. AT1R, angiotensin II receptor 1; Col1α2, collagen I α2; Col3α1, collagen III α1; FN1, fibronectin; IL1β, interleukin 1β; MMP2, metalloproteinase 2; NF-κB, nuclear factor kappa-light-chain-enhancer of activated B cells; TIMPs 1-4, tissue inhibitor of metalloproteinases 1-4; TNFα, tumour necrosis factor α; Vim, vimentin

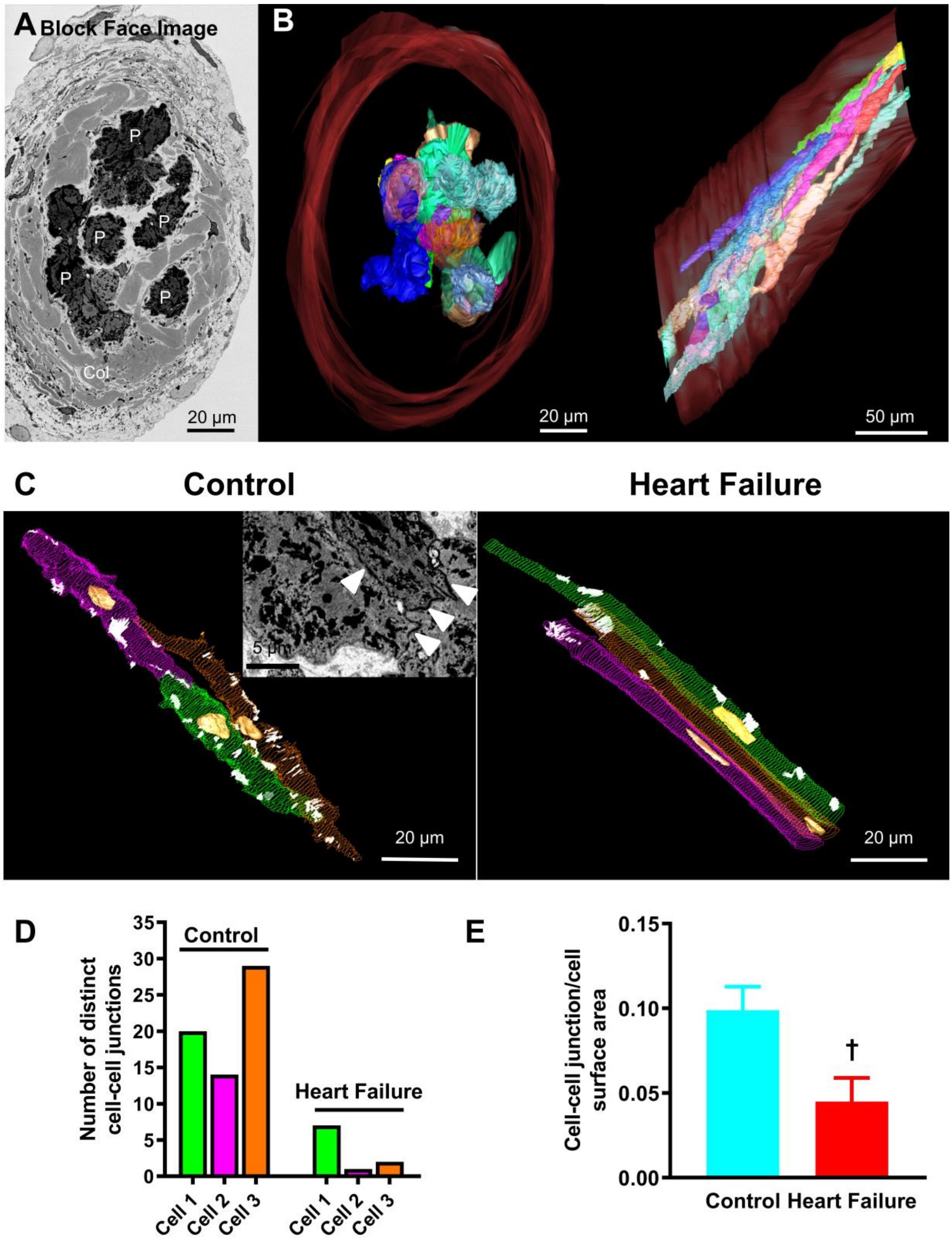

Supplemental Fig. IV. Electron microscopic assessment of left Purkinje fibres. A, Serial block face image of transversely cut control heart free-running left Purkinje fibre. P, Purkinje myocytes; Col, collagen; LCT, loose connective tissue. B, 3D reconstruction of the Purkinje fibre in panel A, shown in cross-section (left) and longitudinally (right). C, 3D reconstruction of individual Purkinje myocytes (green, pink and orange), nuclei (yellow) and cell-cell junctions (white) in control (left) and heart failure (right). Arrowheads in inset denote cell-cell junctions. D, number of distinct cell-cell junctions in the 3D reconstructed myocytes shown in panel C. D, ratio of cell-cell junction surface area to total cell surface area (n=3, †P=0.05)

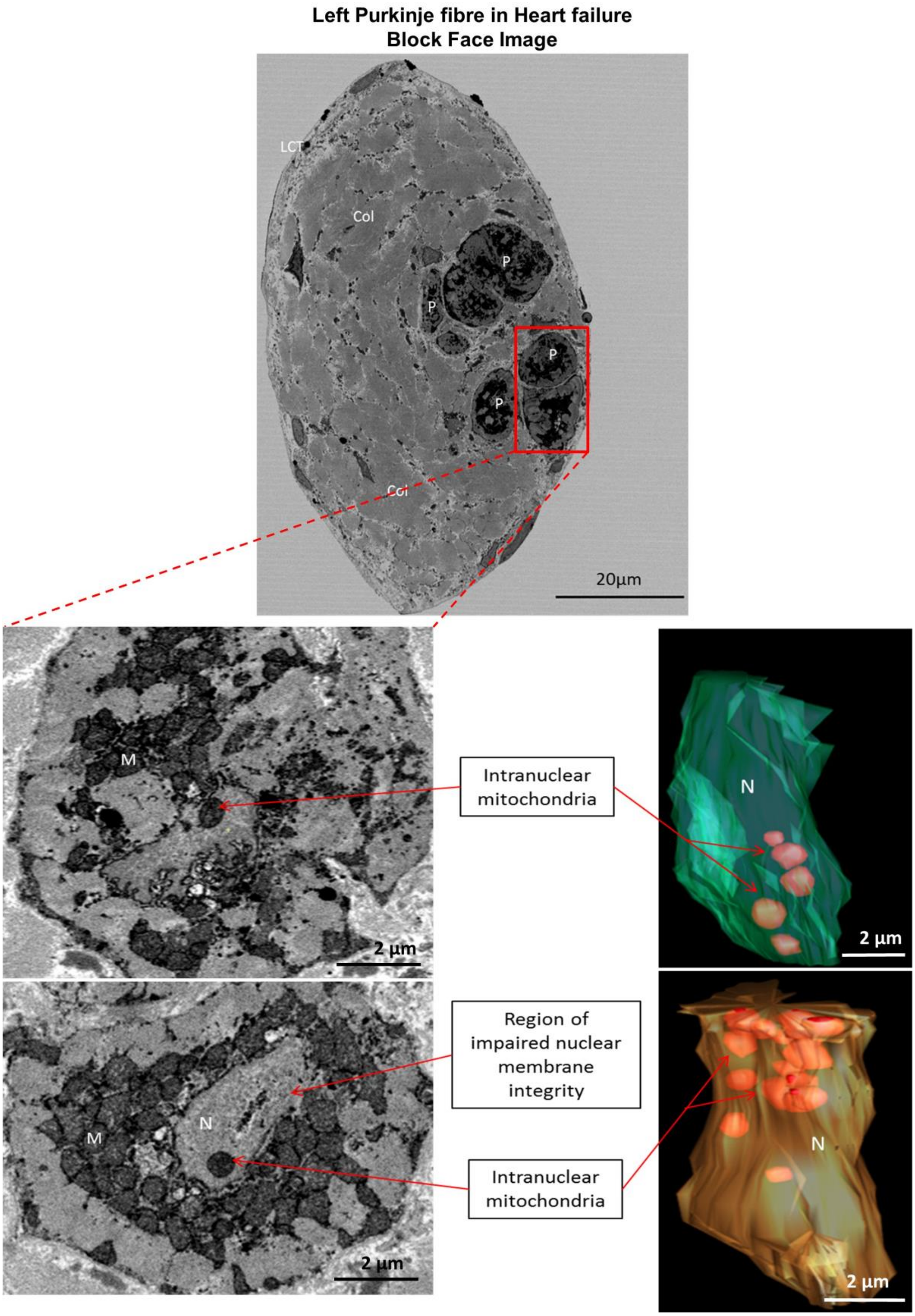

Supplemental Fig. V. Evidence of cellular damage in heart failure. Top, block face image of cross-sectional view of free-running left Purkinje fibre from a failing heart. Below, left, magnified view showing regions of impaired nuclear membrane integrity and internuclear mitochondria. Below, right, 3D visualisations of nuclei shown in left panel.

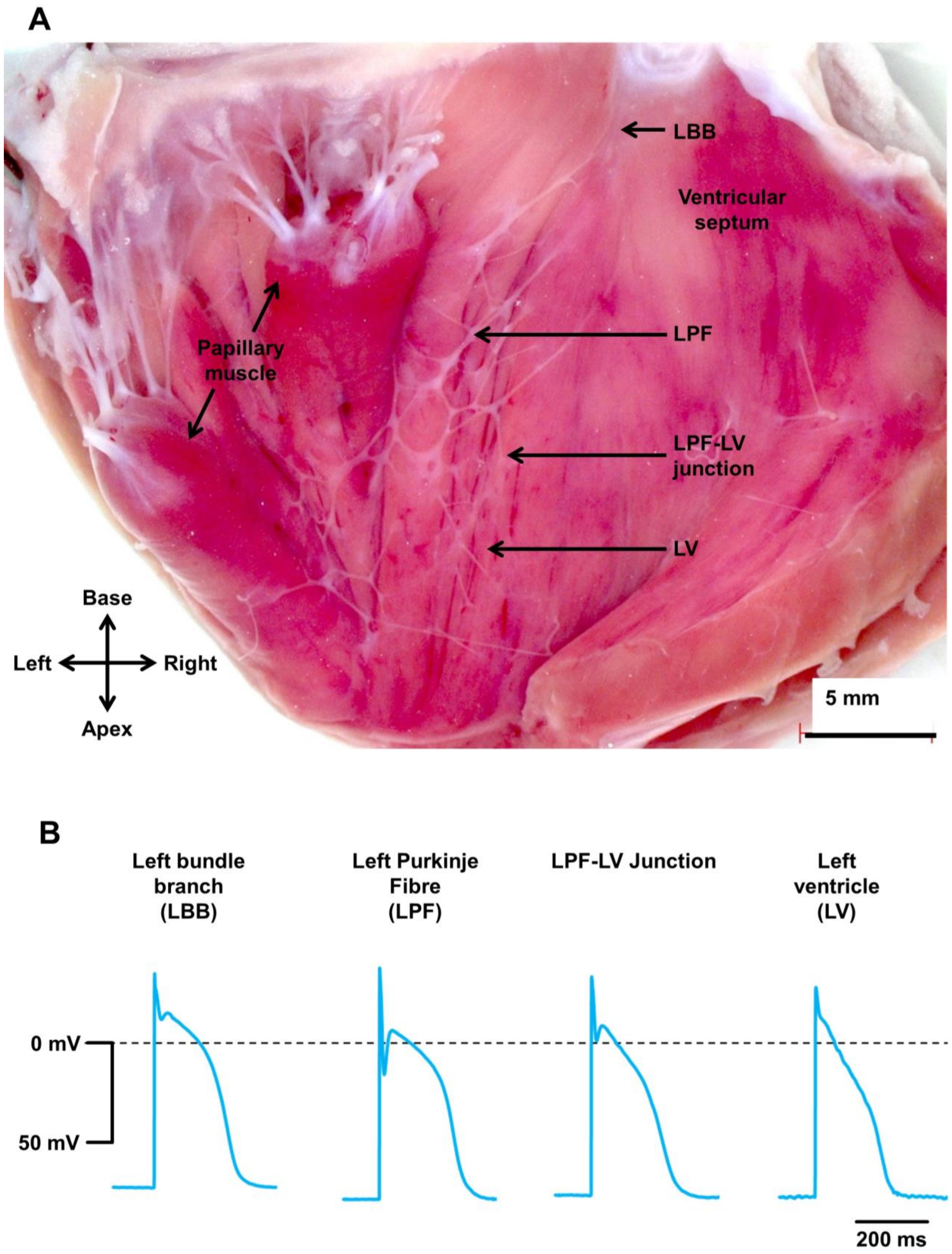

Supplemental Fig. VI. Typical LV preparation used for electrophysiology and action potential recording. A, photograph of a superfused LV preparation used for sharp microelectrode action potential recording. The atria have been removed and the LV opened via a vertical incision through the anterior wall adjacent to the septum. LBB, left bundle branch; FRPF, free-running left PF; JPF, left PF-LV junction. B, representative action potential recordings from the various tissues. LPF, left PF.

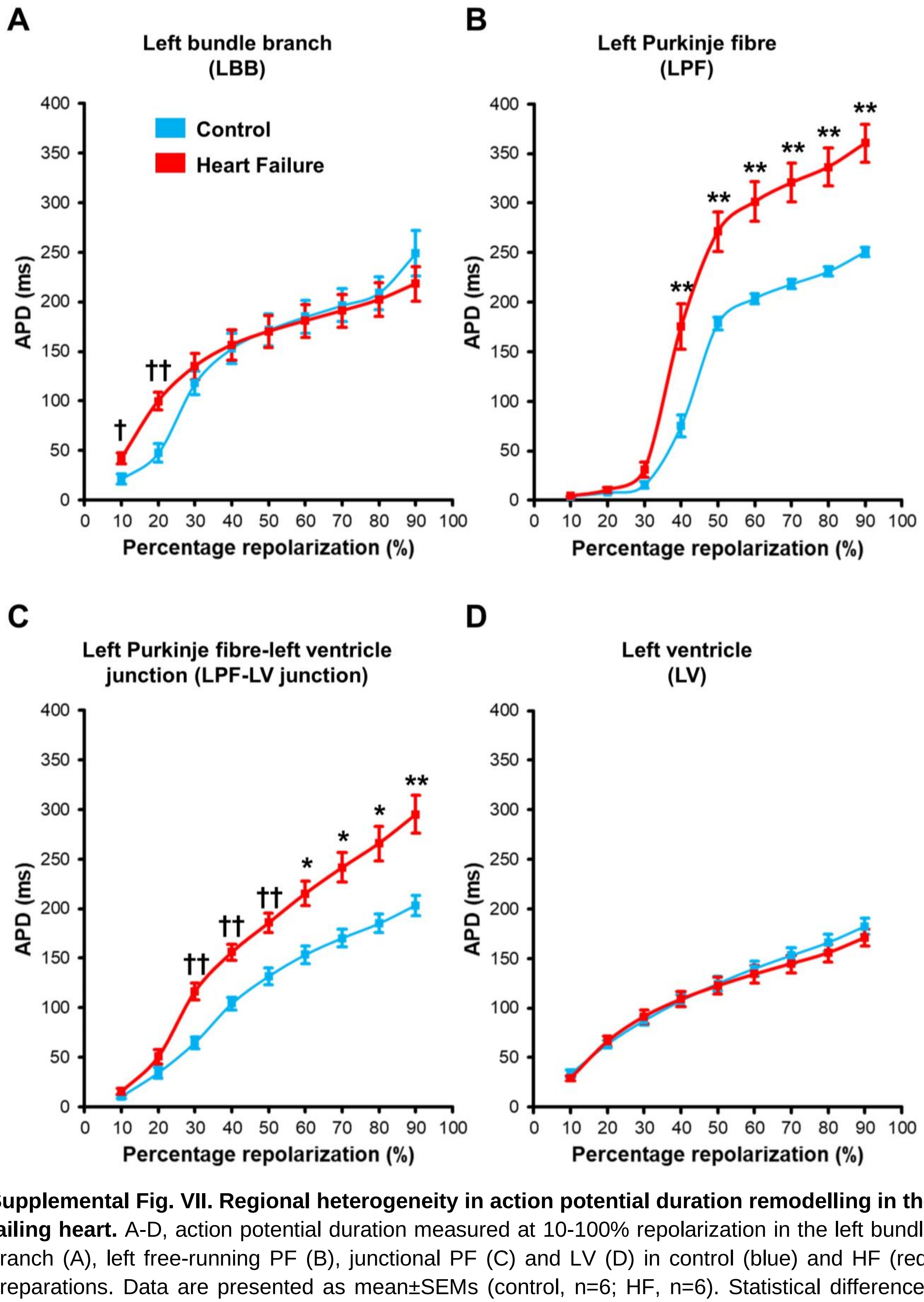

Supplemental Fig. VII. Regional heterogeneity in action potential duration remodelling in the failing heart. A-D, action potential duration measured at 10-100% repolarization in the left bundle branch (A), left free-running PF (B), junctional PF (C) and LV (D) in control (blue) and HF (red) preparations. Data are presented as mean±SEMs (control, n=6; HF, n=6). Statistical differences between control and HF rabbits were assessed using two-way ANOVA (*) and *t*-test (†). *,†*P*≤0.05; **, ††*P*≤0.001.

Supplemental Table I. Quantitative data pertaining to Figures 1 and 2.

Figure	Y-axis parameter	X-axis parameter	Control				Heart Failure (HF)				P values	
			Mean	SD	SEM	n	Mean	SD	SEM	n	Two-way ANOVA adjusted P value	t-test
1B	(g/kg)	Heart/body weight	2.7	0.3	0.1	7	4.2	0.6	0.2	8	0.0287	
		Lung/body weight	3.9	0.5	0.2	7	6.1	2.0	0.7	8	0.0014	
1D	(cm)	LVIDs	0.9	0.1	0.04	7	1.7	0.3	0.1	8	<0.0001	
		LVIDd	1.6	0.3	0.1	7	2.3	0.1	0.04	8	<0.0001	
1E	(%)	Fractional shortening	41.8	2.9	1.1	7	25.3	8.2	2.9	8	0.0002	
		Ejection fraction	79.1	2.4	0.9	7	54.5	10.2	3.6	8	<0.0001	
1F	Cycle length (ms)	Baseline	227.5	39.2	14.8	7	228.3	50.9	18	8	0.9991	
		Autonomic block	242.3	38.6	14.6	7	279.9	27.4	9.7	8	0.1549	0.0466
1G	PR interval (ms)	Baseline	62.2	2.9	1.1	7	70.8	11.1	3.9	8	0.0888	
		Autonomic block	68.3	2.8	1.1	7	83.3	9.8	3.5	8	0.0022	
1H	QRS duration (ms)	Baseline	35.6	4.6	1.7	7	41.5	5.8	2.0	8	0.1279	0.0472
		Autonomic block	38.4	4.3	1.6	7	48.2	8.2	2.9	8	0.0081	
1I	QT_c (ms)	Baseline	248.9	28.1	10.6	7	302.5	16.8	6.0	8	0.0002	
		Autonomic block	281.2	25.2	9.5	7	324.2	18.1	6.4	8	0.0018	
2A	Ventricular tissue volume (mm^3)	LV Free wall	1650.0	398.4	162.6	6	2483.0	753.7	307.7	6	0.0051	
		RV Free wall	760.2	98.1	40.0	6	1120.0	383.1	156.4	6	0.3802	
		IVS	1104.0	233.7	95.4	6	1620.0	340.2	138.9	6	0.1187	0.0119
2B	Ventricular chamber volume (mm^3)	LV	1825.0	811.5	331.3	6	5041.0	1636.0	667.9	6	0.0008	
		RV	2183.0	943.7	385.3	6	2264.0	1644.0	671.2	6	0.993	
2G	Volume (mm^3)	NA	4.2	1.4	0.6	6	11.8	7.4	3.0	6		0.0531
2H	Free running length (mm)	NA	220.6	57.4	23.4	6	359.4	144.1	58.8	6		0.0532
2I	Longest free running length (mm)	NA	5.8	1.1	0.4	6	8.2	0.8	0.3	6		0.001

Supplemental Table II. Changes in gene transcripts in HF in four heart tissues: left and right Purkinje fibres (LPFs and RPFs) and ventricle (LV and RV). Gene categories and individual transcripts are listed in the left-hand columns. Upward and downward arrows indicate significant up- and down regulation in HF in relation to control tissue (n = 6-8 hearts; $P \leq 0.05$). Blank cells denote no change. Proportion of transcripts changing significantly in each tissue type is shown in the last row.

Category	Gene transcripts	LPF	RPF	LV	RV
Ion channels	HNC1	↓	↓		
	HCN4	↓	↓		
	$Na_v1.1$	↓	↑		
	$Na_v1.5$	↓		↑	
	$Ca_v1.2$	↓	↓		
	$Ca_v1.3$	↓			
	$Ca_v3.1$				↑
	$K_v1.4$				
	$K_v1.5$	↓	↓		
	$K_v4.3$		↓		
	KChIP2			↓	
	ERG	↓	↓		
	K_vLQT1	↓			
	minK		↓		
	$K_{ir}2.1$				
	$K_{ir}2.2$	↓	↓		
	$K_{ir}3.1$		↓		
	$K_{ir}6.2$	↓	↓		
	SUR2a	↓	↓	↑	
Gap junction proteins	Cx40	↓			
	Cx43	↓			
Ca^{2+}-handling proteins	NCX1	↓		↑	
	RYR2	↓		↑	
	RYR3	↓	↓		
	SERCA2	↓			
Pump and exchanger	Na/K ATPase α1				
	NHE1				
Others	NFM	↓			
	Tbx3	↓	↓		
	Tbx2				↑
	PAK1	↓	↑		
	β MHC	↓		↑	
	BNP	↓	↓		
	Changed transcripts/total	23/33	16/33	6/33	2/33

Supplemental Table III. Quantitative data pertaining to Figures 3 and 4.

Figure	Y-axis parameter	X-axis parameter	Control				Heart Failure (HF)				P values	
			Mean	SD	SEM	n	Mean	SD	SEM	n	Two-way ANOVA adjusted P value	t-test
3A	HCN1 mRNA	LPF	4.46	2.07	0.92	5	0.56	0.33	0.19	3	<0.0001	
		RPF	2.51	1.47	0.56	7	0.76	0.33	0.14	6	0.0095	
		LV	0.12	0.12	0.05	5	0.27	0.14	0.06	5	0.9986	
		RV	0.24	0.32	0.12	7	0.57	0.45	0.20	5	0.9639	
3B	HCN4 mRNA	LPF	5.90	4.83	1.82	7	1.08	1.52	0.58	7	0.0344	
		RPF	10.44	5.88	2.40	6	4.97	5.06	2.06	6	0.0236	
		LV	0.17	0.16	0.06	8	0.25	0.32	0.14	5	>0.9999	
		RV	1.44	0.86	0.32	7	1.01	0.70	0.32	5	0.999	
3C	Na$_v$1.1 mRNA	LPF	28.81	14.24	8.22	3	9.93	6.25	2.55	6	0.0037	
		RPF	0.00	0.00	0.00	6	9.59	9.00	6.37	2	0.376	0.0187
		LV	1.59	1.87	0.84	5	1.16	1.29	0.65	4	>0.9999	
		RV	6.93	10.63	4.34	6	8.74	0.00	0.00	1	0.9989	
3D	Na$_v$1.5 mRNA	LPF	1.06	0.39	0.15	7	0.51	0.13	0.05	6	0.0714	0.009
		RPF	0.52	0.31	0.12	7	0.66	0.19	0.08	6	0.9471	
		LV	0.08	0.04	0.02	5	0.77	0.91	0.34	7	0.0212	
		RV	0.25	0.14	0.05	8	0.23	0.12	0.05	6	>0.9999	
3E	Ca$_v$1.2 mRNA	LPF	1.55	0.71	0.27	7	0.57	0.19	0.09	5	0.3326	0.0095
		RPF	4.91	2.30	1.15	4	1.37	0.80	0.33	6	<0.0001	
		LV	1.03	1.05	0.37	8	1.03	0.60	0.25	6	>0.9999	
		RV	1.15	0.89	0.40	5	0.35	0.19	0.11	3	0.7267	
3F	Ca$_v$1.3 mRNA	LPF	3.59	0.91	0.52	3	0.35	0.13	0.07	3	0.0012	
		RPF	0.00	0.00	0.00	3	0.42	0.00	0.00	1	0.9887	
		LV	0.28	0.29	0.15	4	1.21	1.56	0.78	4	0.465	
		RV	1.92	1.29	0.91	2	0.00	0.00	0.00	3	0.0996	
3G	Ca$_v$3.1 mRNA	LPF	0.00	0.00	0.00	5	1.44	0.94	0.47	4	0.3357	
		RPF	0.00	0.00	0.00	3	0.00	0.00	0.00	2	>0.9999	
		LV	0.45	0.42	0.29	2	1.04	0.49	0.28	3	0.9758	
		RV	0.00	0.00	0.00	3	4.95	3.31	1.91	3	0.0005	
4A	K$_v$1.4 mRNA	LPF	1.35	1.24	0.62	4	0.00	0.00	0.00	1	0.9729	
		RPF	0.00	0.00	0.00	1	0.00	0.00	0.00	1	>0.9999	
		LV	1.72	0.74	0.30	6	3.35	3.58	1.60	5	0.6782	
		RV	0.85	0.00	0.00	1	0.00	0.00	0.00	1	0.9981	
4B	K$_v$4.3 mRNA	LPF	2.36	1.49	0.74	4	1.28	0.80	0.33	6	0.5912	
		RPF	4.51	2.22	1.28	3	0.57	0.51	0.30	3	0.0034	
		LV	0.85	0.59	0.21	8	0.46	0.34	0.15	5	0.9735	
		RV	2.39	2.67	1.34	4	0.22	0.07	0.05	2	0.228	

Supplemental Table IV. Quantitative data pertaining to Figure 4.

Figure	Y-axis parameter	X-axis parameter	Control				Heart Failure (HF)				P values	
			Mean	SD	SEM	n	Mean	SD	SEM	n	Two-way ANOVA adjusted P value	t-test
4C	KChIP2 mRNA	LPF	0.00	0.00	0.00	3	0.01	0.00	0.00	3	>0.9999	
		RPF	0.00	0.00	0.00	3	0.01	0.00	0.00	1	>0.9999	
		LV	0.33	0.36	0.13	8	0.02	0.00	0.00	4	0.1266	0.0437
		RV	0.30	0.22	0.08	7	0.15	0.08	0.05	3	0.8363	
4D	K_v1.5 mRNA	LPF	2.00	0.99	0.38	7	0.39	0.22	0.08	7	0.0006	
		RPF	2.44	1.15	0.51	5	0.93	0.63	0.28	5	0.0079	
		LV	0.90	0.86	0.31	8	0.73	0.53	0.20	7	0.9861	
		RV	0.99	0.69	0.26	7	0.59	0.29	0.12	6	0.7994	
4E	ERG mRNA	LPF	1.17	0.82	0.37	5	0.28	0.09	0.04	6	0.0465	
		RPF	1.85	1.50	0.86	3	0.50	0.28	0.14	4	0.0112	
		LV	0.09	0.08	0.03	5	0.65	0.67	0.25	7	0.3099	
		RV	0.35	0.21	0.08	7	0.42	0.13	0.05	6	0.999	
4F	K_vLQT1 mRNA	LPF	4.90	3.71	1.52	6	0.92	0.78	0.32	6	0.0007	
		RPF	4.22	2.01	1.42	2	1.76	1.30	0.58	5	0.3007	
		LV	1.21	0.87	0.31	8	1.91	1.27	0.48	7	0.8871	
		RV	2.27	0.88	0.33	7	1.56	0.87	0.39	5	0.9239	
4G	minK mRNA	LPF	5.00	5.75	2.35	6	2.25	1.02	0.39	7	0.9083	
		RPF	21.02	22.34	12.90	3	5.77	5.34	3.09	3	0.0272	
		LV	0.73	0.52	0.18	8	3.24	4.04	2.02	4	0.9514	
		RV	0.85	1.06	0.53	4	2.44	1.70	0.85	4	0.9947	
4H	K_{ir}2.1 mRNA	LPF	0.55	0.48	0.20	6	0.12	0.13	0.05	8	0.2392	
		RPF	0.73	0.24	0.10	6	0.55	0.60	0.23	7	0.9116	
		LV	0.12	0.04	0.02	5	0.61	0.63	0.24	7	0.1811	
		RV	0.49	0.49	0.18	7	0.30	0.15	0.06	6	0.889	
4I	K_{ir}2.2 mRNA	LPF	8.53	4.28	2.14	4	0.26	0.04	0.02	3	<0.0001	
		RPF	8.44	0.00	0.00	1	0.71	0.69	0.49	2	0.0087	
		LV	0.53	0.35	0.12	8	0.94	0.58	0.26	5	0.9912	
		RV	1.85	1.33	0.77	3	2.62	1.94	1.12	3	0.976	
4J	K_{ir}3.1 mRNA	LPF	4.36	4.52	2.02	5	0.29	0.12	0.06	5		
		RPF	4.94	1.42	1.00	2	0.29	0.17	0.12	2		0.0438
		LV	0.18	0.09	0.05	3	0.42	0.57	0.33	3		
		RV	0.53	0.53	0.37	2	Technical error					
4K	K_{ir}6.2 mRNA	LPF	1.50	0.87	0.36	6	0.13	0.06	0.03	5	0.0043	
		RPF	3.36	1.73	1.00	3	0.30	0.16	0.09	3	<0.0001	
		LV	0.31	0.40	0.16	6	0.44	0.36	0.14	7	0.9923	
		RV	0.73	0.56	0.21	7	0.43	0.39	0.16	6	0.8715	

Supplemental Table V. Quantitative data pertaining to Figures 4 and 5.

Figure	Y-axis parameter	X-axis parameter	Control				Heart Failure (HF)				P values	
			Mean	SD	SEM	n	Mean	SD	SEM	n	Two-way ANOVA adjusted P value	t-test
4L	SUR2a mRNA	LPF	0.41	0.16	0.06	6	0.21	0.10	0.04	7	0.3068	0.0175
		RPF	0.73	0.48	0.28	3	0.25	0.11	0.06	4	0.014	
		LV	0.06	0.03	0.01	5	0.40	0.34	0.13	7	0.032	
		RV	0.17	0.10	0.04	7	0.26	0.12	0.05	5	0.9194	
5A	SERCA2a mRNA	LPF	0.25	0.12	0.04	8	0.10	0.04	0.01	7	0.2757	0.0088
		RPF	0.25	0.15	0.06	6	0.26	0.22	0.09	6	>0.9999	
		LV	0.06	0.01	0.00	5	0.23	0.20	0.07	7	0.3105	
		RV	0.29	0.28	0.10	7	0.11	0.09	0.04	6	0.2027	
5B	RyR2 mRNA	LPF	0.35	0.21	0.08	7	0.15	0.12	0.04	7	0.5506	0.0499
		RPF	0.28	0.09	0.04	6	0.35	0.27	0.10	7	0.9852	
		LV	0.25	0.23	0.09	6	0.74	0.59	0.22	7	0.0088	
		RV	0.10	0.03	0.01	6	0.18	0.10	0.04	6	0.9805	
5C	RyR3 mRNA	LPF	3.32	1.45	1.02	2	0.58	0.36	0.15	6	0.0001	
		RPF	1.77	0.00	0.00	2	0.22	0.06	0.04	2	0.0997	0.0008
		LV	0.41	0.26	0.09	8	0.97	1.11	0.50	5	0.4703	
		RV	1.02	1.12	0.64	3	0.00	0.00	0.00	5	0.1576	
5D	NCX1 mRNA	LPF	0.32	0.07	0.03	7	0.15	0.08	0.03	7	0.2263	0.0008
		RPF	0.29	0.18	0.07	6	0.25	0.11	0.05	5	0.9907	
		LV	0.07	0.03	0.01	5	0.45	0.39	0.15	7	0.0019	
		RV	0.11	0.07	0.03	6	0.11	0.06	0.02	7	>0.9999	
5E	Na^+/K^+-ATPase mRNA	LPF	0.73	0.32	0.12	7	0.50	0.20	0.08	7	0.5061	
		RPF	0.58	0.13	0.06	5	0.53	0.39	0.16	6	0.9978	
		LV	0.08	0.02	0.01	5	0.48	0.52	0.20	7	0.106	
		RV	0.29	0.24	0.09	7	0.21	0.12	0.05	6	0.9819	
5F	Na^+/H^+-exchanger mRNA	LPF	1.80	0.93	0.35	7	0.94	0.91	0.34	7	0.2434	
		RPF	2.54	1.43	0.72	4	1.81	1.32	0.54	6	0.5863	
		LV	0.23	0.15	0.06	7	0.36	0.19	0.09	4	0.9988	
		RV	0.44	0.26	0.11	6	1.08	0.81	0.36	5	0.6421	
5G	Cx40 mRNA	LPF	1.10	0.59	0.21	8	0.55	0.21	0.08	7	0.4833	0.0371
		RPF	1.45	0.99	0.41	6	1.59	1.65	0.67	6	0.9953	
		LV	0.26	0.22	0.08	8	0.57	0.41	0.16	7	0.887	
		RV	0.42	0.26	0.11	6	0.42	0.36	0.16	5	>0.9999	
5H	Cx43 mRNA	LPF	1.28	0.61	0.23	7	0.66	0.34	0.12	8	0.0391	
		RPF	0.95	0.54	0.20	7	1.02	0.57	0.23	6	0.9976	
		LV	0.13	0.04	0.02	5	0.60	0.57	0.21	7	0.2931	
		RV	0.41	0.33	0.12	8	0.28	0.14	0.06	6	0.9724	

Supplemental Table VI. Quantitative data pertaining to Figures 6-8.

Figure	Y-axis parameter	X-axis parameter	Control				Heart Failure (HF)				P values	
			Mean	SD	SEM	n	Mean	SD	SEM	n	Two-way ANOVA adjusted P value	t-test
6B	Myocyte diameter (µm)	LPF	14.9	4.2	0.4	91	19.3	4.1	0.3	161	<0.0001	
		RPF	15.5	3.5	0.4	91	14.7	4.0	0.3	152	0.5263	
		LV	16.7	3.7	0.4	79	20.9	5.3	0.5	111	<0.0001	
		RV	15.5	3.3	0.4	83	18.5	5.8	0.5	119	<0.0001	
6C	Cx40 Intensity (A.U.)	LPF	6.6	0.4	0.2	3	8.3	1.2	0.5	5	0.07	
		LV	6.6	0.7	0.4	3	8.1	1.1	0.5	5	0.1085	
6D	Cx43 Intensity (A.U.)	LPF	42.1	9.7	5.6	3	32.1	1.0	0.5	5	0.0233	
		LV	48.3	3.1	1.8	3	51.7	3.3	1.5	5	0.5553	
7B	Ventricular rate (bpm)	LPF	89.2	31.4	5.1	38	87.5	28.1	5.9	23	0.9747	
		LV	84.9	41.9	9.9	18	82.9	30.8	6.3	24	0.9747	
7C	RMP (mV)	LPF	-79.0	4.5	0.7	38	-84.5	5.8	1.2	23	0.0003	
		LV	-78.8	4.4	0.9	22	-80.1	6.7	1.4	24	0.638	
7D	dV/dtmax (V/s)	LPF	316.0	68.3	11.1	38	266.0	96.2	20.1	23	0.0217	
		LV	159.0	70.9	16.7	18	208.0	53.2	10.9	24	0.0661	0.0143
7E	Amplitude (ms)	LPF	112.6	7.4	1.2	38	114.2	7.2	1.5	23	0.6034	
		LV	107.2	5.7	1.3	18	112.8	5.8	1.2	24	0.0184	
7F	APD_{10} (ms)	LPF	4.0	0.9	0.1	38	4.5	1.2	0.2	23	0.9748	
		LV	33.7	15.1	3.6	18	29.2	14.3	2.9	24	0.2351	
7G	APD_{50} (ms)	LPF	179.2	45.5	7.4	38	300.1	109.1	22.7	23	<0.0001	
		LV	124.5	29.8	7.0	18	122.3	41.5	8.5	24	0.9919	
7H	APD_{90} (ms)	LPF	248.6	31.4	5.1	38	377.7	116.6	24.3	23	<0.0001	
		LV	182.2	33.6	7.9	18	170.8	41.6	8.5	24	0.8101	
8A	APD_{50} (ms)	LBB	171.7	53.5	16.1	11	170.0	56.2	16.2	12	>0.9999	
		LPF	179.2	45.5	7.4	38	300.1	109.1	22.7	23	<0.0001	
		LPF-LV Junction	131.5	27.4	8.3	11	185.6	55.2	9.6	33	0.0358	
		LV	124.5	29.8	7.0	18	122.3	41.5	8.5	24	>0.9999	
8B	APD_{90} (ms)	LBB	249.0	77.1	23.3	11	218.1	60.9	17.6	12	0.7867	
		LPF	248.6	31.4	5.1	38	377.7	116.6	24.3	23	<0.0001	
		LPF-LV Junction	203.4	33.8	10.2	11	294.9	109.7	19.1	33	0.0021	
		LV	182.2	33.6	7.9	18	170.8	41.6	8.5	24	0.9798	

Supplemental Table VII. Left and right primer sequences for rabbit pro-inflammatory and fibrotic genes of interest used for qPCR analysis.

Gene	Primer	Sequence (5′→3′)	Reference sequence
Interleukin 1 beta (IL1β)	Left	CTCTCCAGCCACTCTTCATTGTT	NM_001082201.1
	Right	CCACTGTGGTAAGCCATCATCT	
Tumor necrosis factor (TNF)	Left	TCTGCCTCAGCCTCTTCTCTTT	NM_001082263.1
	Right	AGGGTTGACTAGATGGAGGTTGTTT	
Nuclear factor kappa-light-chain-enhancer of activated B cells (NFκB)	Left	GGATTTCGTTTCCGTTATGTCTG	ENSOCUT00000027001
	Right	TGAGGGTAGGACTTCTTGTTCTTC	
Angiotensin II receptor type 1 (ATR1)	Left	TCAGAAGAACAAGCCAAGAAATG	NM_001082324.1
	Right	GGAACCCAGGAGAAGAAAAAGAA	
Collagen type I alpha 2 (Col1α2)	Left	AATCACGCCTCTCAGAACATCAC	NM_001195668.1
	Right	TTGTTCAAGTTGCCAGTTTCCTC	
Collagen type III alpha 1 (Col3α1)	Left	TCCTGGGAAAGTCCACAAGTAAA	XM_002712333.3
	Right	GAAAGGATCGAACCACATACAAG	
Fibronectin-1 (FN1)	Left	ACCTTCCAGAAACTGCCAACTC	XM_002712573.1
	Right	GCACTCTCTTGGTTTTCTTCCAC	
Vimentin (Vim)	Left	CTGCCTCTTCCAAACTTTTCTTCT	XM_002717420.1
	Right	AAGTGTCCTTTTTGAGTGGGTGT	
Matrix metalloproteinase-2 (MMP2)	Left	TCAAGTGGTCCGTGTGAAGTATG	NM_001082209.1
	Right	CTTCTCAAAGTTGTAGGTGGTGGA	
Tissue inhibitor of metalloproteinases 1 (TIMP1)	Left	AAAGACTGAAGGCTGCTCCTGTT	NM_001082232.2
	Right	GACAAAGAAAGATGGGGGTAGGA	
Tissue inhibitor of metalloproteinases 2 (TIMP2)	Left	ATGATCCCGTGCTACATCTCCT	XM_002723776.1
	Right	TGCCCGTTGATGTTCTTCTC	
Tissue inhibitor of metalloproteinases 3 (TIMP3)	Left	CTTCGTGACCTCCAAGAATGAGT	NM_001195682.1
	Right	CAGGCATAGTGTTTGGACTGGTAG	
Tissue inhibitor of metalloproteinases 4 (TIMP4)	Left	AGTTAGAAGCCAACAGCCAGAAG	NM_001195690.1
	Right	GATGTAGTTGCACAAATGAATGAAG	
